# Rare earth smart nanomaterials for bone tissue engineering and implantology: Advances, challenges, and prospects

**DOI:** 10.1002/btm2.10262

**Published:** 2021-12-01

**Authors:** Duraipandy Natarajan, Zhitong Ye, Liping Wang, Linhu Ge, Janak Lal Pathak

**Affiliations:** ^1^ Affiliated Stomatology Hospital of Guangzhou Medical University Guangdong Engineering Research Center of Oral Restoration and Reconstruction, Guangzhou Key Laboratory of Basic and Applied Research of Oral Regenerative Medicine Guangzhou China

**Keywords:** bone grafts, bone tissue engineering, implantology, nanomaterials, RE materials

## Abstract

Bone grafts or prosthetic implant designing for clinical application is challenging due to the complexity of integrated physiological processes. The revolutionary advances of nanotechnology in the biomaterial field expedite and endorse the current unresolved complexity in functional bone graft and implant design. Rare earth (RE) materials are emerging biomaterials in tissue engineering due to their unique biocompatibility, fluorescence upconversion, antimicrobial, antioxidants, and anti‐inflammatory properties. Researchers have developed various RE smart nano‐biomaterials for bone tissue engineering and implantology applications in the past two decades. Furthermore, researchers have explored the molecular mechanisms of RE material‐mediated tissue regeneration. Recent advances in biomedical applications of micro or nano‐scale RE materials have provided a foundation for developing novel, cost‐effective bone tissue engineering strategies. This review attempted to provide an overview of RE nanomaterials' technological innovations in bone tissue engineering and implantology and summarized the osteogenic, angiogenic, immunomodulatory, antioxidant, in vivo bone tissue imaging, and antimicrobial properties of various RE nanomaterials, as well as the molecular mechanisms involved in these biological events. Further, we extend to discuss the challenges and prospects of RE smart nano‐biomaterials in the field of bone tissue engineering and implantology.

## 1 INTRODUCTION

Rare earth (RE) materials are found naturally in a thin layer of earth surfaces.^1^, ^2^, ^3^ RE metals are found in the ores like basalts, granites, gneisses, shales, clays, and silicate rocks. Yttrium and lanthanides are the commonly known RE metals. The Finnish chemist Johan Gadolin isolated the first RE element yttrium in 1794 from gadolinite near Ytterby (Sweden). Seventeen lanthanides have been identified so far.^4^ Among lanthanides, cerium is the most abundant element (60–68 ppm), followed by neodymium and lanthanum.^5^, ^6^ Praseodymium, samarium, gadolinium (Gd), and dysprosium have abundances in the range of 5–10 ppm, while other elements are less abundant, with lutetium being the least abundant (<0.5 ppm).^7^ The electronic configuration of RE elements is ([Xe]4f^*n*^5s^2^5p^6^ [*n* = 0–14]) and usually exists as trivalent cations. The outer 5s substantially shield the 4f electrons and 5p electrons, and hence the electronic transitions from 4f to 4f or from 4f to 5d are barely affected by the surrounding environment. Therefore, the RE materials have sufficient energy levels and several unique spectroscopic characters such as extended lifetime emission and narrow bandwidth with sharp fluorescent emissions via photoluminescence.^8^, ^9^ Generally, photoluminescence obeys Stokes law that means the wavelength of the emitted fluorescence light is more extended than incident light, termed the “downconversion” luminescence. Downconversion luminescence converts higher‐energy photons into lower‐energy photons. For instance, ultraviolet (UV) radiation excites Eu^3+^, Tb^3+^, and Dy^3^ and emits in the visible region. UV excitation of Nd^3+^ emits in the near‐infrared (NIR) region. Excitation by long‐wavelength radiation (i.e., anti‐Stokes luminescence) of Er^3+^ or Tm^3+^ emits shorter‐wavelength light. The emitted fluorescence light is in a shorter wavelength and higher energy than the incident light; thereby, it is called anti‐Stokes luminescence or “upconversion” luminescence. Therefore, RE materials are gaining their significance in biomedical imaging owing to the reduction of autofluorescence and penetrating properties in the tissues of biological systems.^10^, ^11^, ^12^

Various electronic configurations and variable valence states are crucial in enhancing the stability, broadening the absorption range endowed RE ions with flexible redox properties and unique luminous and electromagnetic characteristics.^13^, ^14^, ^15^ These properties of RE elements attribute to the design of nanostructured materials either as major components or as dopants paving the way for new tissue engineering applications. The particle size ranging from 1 to 100 nm of nanoparticles and geometry has been reported to play an essential role in cell–material interactions, affecting cellular uptake, and cell functioning.^16^ Most cell–nanoparticle interactions have been facilitated at nano biointerface by several factors such as nanoparticle's shape and surface morphology.^16^ The shape/geometry of the nanoparticles directly influences their cellular uptake. It has been observed that rod‐shaped particles have the highest uptake, followed by spheres, cylinders, and cubes.^17^ Similarly, the neodymium nanoparticle's shape influences the cellular activity in terms of altered mitochondrial membrane potential, reactive oxygen species (ROS), and eventually angiogenesis in endothelial cells.^18^ The cellular uptake of nanomaterials such as liposomes,^19^ iron oxide,^20^ polymeric,^21^ gold,^22^, ^23^, ^24^ and silica nanoparticles^25^ is size dependent. The particle size of the polystyrene spheres increased the binding and affected the immune response in human dendritic cells.^26^ Similarly, the RE materials like ceria have the highest cellular uptake and reactive oxygen species production in human monocyte cell line U937,^27^ size dependence cell viability in Hela and HEK cells,^28^ and size dependence biodistribution of ceria was also observed in rat animal model.^28^ Further, rare‐earth fluorides such as erbium showed good cell imaging features depends on their size.^29^ Besides that, many factors, such as surface chemistry and oxidation states of RE metals like ceria, affected the physiological conditions.^30^ Few studies reported that RE materials doped mesoporous silica nanoparticle and polymeric nanoparticles possess positively charged that could be facilitated the cell nanomaterial interactions.^21^

Moreover, in vivo assay usually demands controlled particle size to use the enhanced permeation and retention effect, high colloidal stability, and low toxicity.^31^ RE metal‐based nanoparticles are used in different imaging approaches other than luminescent imaging like magnetic resonance imaging (MRI) and computed tomography (CT).^32^ RE materials hold a robust therapeutic potential owing to biocompatibility, optical, and physicochemical properties. Lanthanides are widely used in the electronic and painting industry due to their magnetic and adsorption properties.^33^, ^34^ The magnetic properties of some lanthanide cations such as Gd^3+^, Ho^3+^, and Dy^3+^ make RE‐based nanoparticles of these cations very useful in MRI because these cations can induce additional contrast between normal and abnormal regions.^35^, ^36^ In the biological field, various functions of RE elements have been reported. Recently, researchers have been trying to use the intrinsic optical properties of RE nanomaterials for in vivo imaging to monitor the physiologic processes.^37^, ^38^, ^39^ Besides that, in compliance with unique features, these materials are used for in situ bio‐labeling of cellular organelles, photodynamic therapy in tumor targeting, site‐specific delivery of therapeutic molecules with a combination of fluorescence and the therapeutic effect as a theranostic tool.^40^, ^41^, ^42^, ^43^, ^44^ Due to the high adsorbing affinity, RE has been widely used as a doping material with metal to produce alloy materials for bone and dental prostheses production.^45^, ^46^ RE nanoparticles can be incorporated into the connectivity centers or inside the metal–organic frameworks.^35^, ^47^ Highly porous and oriented structures allow RE nanoparticles to accommodate many different functional carrier cargoes like drugs, growth factors and make them attractive materials for biomedical applications.^33^ The development of RE‐based smart nano‐biomaterials with osteogenic, angiogenic, and immunomodulatory potential and in vivo imaging has a massive scope in the field of bone tissue engineering and implantology. Significant advancements have been made with RE in bone grafts and prostheses design in the past two decades. Here, we have listed the advances and potential applications of these RE smart nano‐biomaterials in bone tissue engineering and implantology.

## 2 BONE CELL BIOLOGY

Bone is a metabolically growing vital organ that gives the body structural (mechanical stability) and functional properties. The bone progenitor cells carry out different functions such as bone formation, resorption, repair, and mineral homeostasis. The bone progenitor cells originate from two cell lineages, mesenchymal and hematopoietic.^48^ Osteoblasts and osteocytes are differentiated from the mesenchymal stem cells (MSCs). Bone marrow mononuclear hematopoietic cells differentiate into osteoclasts.^49^, ^50^, ^51^ Osteoclasts resorb old and defected bone matrix, and osteoblasts deposit new bone matrix in that place. This phenomenon is called bone remodeling.^52^, ^53^, ^54^, ^55^, ^56^, ^57^, ^58^, ^59^ Balanced osteoblast and osteoclast activity maintain healthy bone.^60^ Certain pathological conditions disrupt the osteoblast and osteoclast function, causing bone loss or excessive bone mass.^61^ Osteocytes are embedded in the bone matrix, comprise 95% of cells in bone, and have the most extended half‐life (25 years) among the bone cells.^62^ A bone matrix consists of organic and inorganic components. The inorganic matrix, calcium, phosphorus, sodium, and magnesium are associated with bone mineral crystals. Bone mineral crystals have shown in the form of apatite, hydroxyapatite (HA), (CaO[PO_4_][OH_2_), and acid phosphate groups (HPO_4_)^2^ containing brushite (CaHPO^2^_4_2H_2_O). These minerals serve as an ion reservoir, which helps maintain their extracellular fluid concentrations for critical physiological functions and gives stiffness and strength to the bone.^63^

Osteocytes sense biological and mechanical stimuli and produce a range of signaling molecules to control osteoblast^64^, ^65^, ^66^, ^67^ and osteoclast functions.^68^, ^69^, ^70^ The balanced function of osteoblasts, osteoclasts, and osteocytes is vital for effective bone regeneration and implant success.^60^, ^71^, ^72^ Researchers are currently developing bone‐biomaterials and implants that can modulate osteoblast, osteoclast, or osteocyte function.^73^, ^74^, ^75^ RE materials have shown the potential to modulate osteoblast, osteoclast, or osteocyte function.^76^, ^77^, ^78^, ^79^ Therefore, RE‐based bone‐biomaterials and implants could be the next generation bone graft, implant, and prosthetics for effective bone tissue engineering.

Besides these, endothelial cells, immune cells, and neuronal cells regulate bone regeneration and homeostasis. These cells produce various signaling molecules affecting bone cells' functions in an autocrine or paracrine manner. Endothelial cells influence bone formation through neovessel formation and release of various growth factors needed for osteogenic differentiation of precursor cells. The endothelial cells lie nearby the bone cells and secrete growth factors like platelet‐derived growth factor (PDGF)‐BB and vascular endothelial growth factor (VEGF) to promote osteogenic differentiation of precursor cells.^80^, ^81^ Moreover, neovasculogenesis is crucial to supply oxygen and growth factors for the precursor cells migrated to the defect sites. Osteogenic cell‐secreted osteopontin induces early angiogenesis in developing bone.^82^, ^83^ The immune cells, including monocytes, neutrophils, dendritic cells, and B and T lymphocytes, play a vital role in osteoimmunomodulation. Biomaterial‐mediated M1 and M2 polarization of macrophages regulate different stages of bone defect healing.^84^, ^85^ The key molecules responsible for the signaling between osteoclasts and osteoblasts are regulated by immune cells.^86^, ^87^, ^88^ The immune cell‐secreted tumor necrosis factor‐α (TNF‐α), interleukin (IL)‐6, and IL‐1β enhance osteoclast differentiation and bone resorption via receptor activator for nuclear factor‐κB ligand (RANKL) secretion.^89^ These pro‐inflammatory cytokines inhibit osteoblast differentiation.^89^ Whereas anti‐inflammatory cytokines, including IL‐4 and IL‐10, increase bone formation by inducing osteoblast function and inhibiting osteoclastogenesis.^90^ Chen et al. have summarized the biomaterial–immune cell interaction and its effect on bone defect healing and osseointegration.^84^, ^85^ Their review suggested the development of novel biomaterials with osteoimmunomodulatory properties for orthopedic and dental applications. Reports from literature had shown the immunomodulatory potential of RE materials,^91^, ^92^ which is thoroughly discussed in Section 5.1.2 of this review.

Neuronal cells also significantly contribute to maintaining skeletal homeostasis. The bone marrow consists of the sympathetic nervous system (SNS) and parasympathetic nervous system (PNS). SNS closely associates with the blood vessels through the nutrient foramen and innervating different regions; some nerves reach bone marrow and connect with transcortical vessels in the bone.^93^ Further, neuron regulates various hematopoietic cell functions via neurotransmitters' binding to beta‐adrenergic receptors.^94^ The PNS may innervate the distal femoral metaphysis and uses acetylcholine as the primary neurotransmitter, which binds to muscarinic or nicotinic receptors.^95^ Apart from the direct regulation of hematopoietic cells, PNS regulates bone remodeling.^96^, ^97^, ^98^, ^99^ Implant‐derived magnesium has been reported to promote bone healing via local neuronal production of calcitonin gene‐related polypeptide‐α (CGRP).^100^ RE element Gd‐doped magnesium scaffold has been reported to enhance bone defect healing via neuronal CGRP‐mediated effect on osteogenesis and angiogenesis.^101^ These findings further strengthen the scope of RE‐based biomaterials in orthopedics and implantology.

## 3 NANOMATERIALS AND CELLS INVOLVED IN BONE REGENERATION

The unprecedented pathological or congenital malfunctions affect bone metabolism by aberrant or restricted actions of the aforementioned bone cells. Thereby understanding the pathophysiology of these cells cues the novel therapeutic targets for bone‐related diseases. Many therapeutic strategies have been developed like small molecules, recombinant proteins, peptides, and plant‐based phytochemicals to eliminate bone therapy‐related complications. Recently, the role of nanoparticles has significantly compromised the need for bone therapeutics. The organic and inorganic components of the bone matrix directly facilitate bone regeneration and maintain bone homeostasis. RE nanomaterials can be designed in combination with organic and inorganic components of the bone matrix to improve bone regeneration. Various metal ions, including RE, had been reported to modulate the osteocyte, osteoblast, and osteoclast activity. Gold nanoparticles incorporated gelatin hydrogels promote proliferation and differentiation of human adipose‐derived stem cells toward osteoblast cells in a dose‐dependent manner.^102^ Another study indicated that the gold nanoparticles suppress osteoclast formation in a dose‐dependent manner and increase bone density that can be useful in preventing and treating osteoporosis.^103^ The gold nanoparticle‐labeled MSCs improve contrast for imaging, and gold nanoparticles preserve the migratory capacity of MSCs.^104^ The gold nanoparticle‐functionalized mesoporous silica nanoparticles synergistically increase the immunomodulatory effects and direct osteogenic stimulation by increasing the osteogenic differentiation capability of MC3T3‐E1 cells and accelerate new bone formation in a critical‐sized cranial defect site in rats.^105^ The therapeutic potential of Ag–Au–HA compositions would be excellent for bone regeneration and fracture healing.^106^ Surface modification of bone grafts with silver nanoparticles, samarium, and TiO_2_ prevents the risk of contamination and infection in alveolar bone and dental implant surgery.^107^, ^108^ The iron oxide nanoparticles coated with dextrin and chitosan increase osteoblast proliferation and differentiation.^109^, ^110^ The inorganic nanoparticles like calcium phosphate nanoparticles increase the osteogenic differentiation of rat bone marrow stromal cells,^111^, ^112^, ^113^ and magnesium‐containing biocomposites facilitate femur fracture repair.^100^, ^114^, ^115^, ^116^, ^117^, ^118^, ^119^, ^120^, ^121^ In this pipeline, the RE nanoparticles have tremendous potential for bone graft development since it has versatile bio applications, including an antioxidant to antimicrobial effect.^122^, ^123^, ^124^ Furthermore, RE metals can be doped in the abovementioned nanoparticles to redevelop the smart nano‐biomaterials with improved antimicrobial, immunomodulatory potential of ceria,^125^, ^126^ the osteo‐angiogenic effect of europium,^127^, ^128^, ^129^ contrast imaging potential of Gd,^130^, ^131^ and laser irradiation property of neodymium.^132^, ^133^ This review exemplifies the role of various RE nanomaterials for the therapeutic modulation of these important bone cells.

## 4 BONE DEFECT HEALING

Critical size or large bone defects need medical interventions to restore.^134^, ^135^, ^136^ A typical bone defect repair consists of four overlapping stages: the initial inflammatory response, soft callus formation, hard callus formation, and bone remodeling.^137^, ^138^ Bone defect healing starts with an initial anabolic phase, where local tissue volume increases through inflammation, and hematoma is formed at the defect site immediately.^139^, ^140^ It has been reported that mesoporous silica nanoparticles, silver and gold nanoparticles can induce inflammation, activating the inflammatory cascades to recruit endothelial cells and neutrophils. Ceria nanoparticles and europium‐doped mesoporous silica nanospheres (Eu‐MSNs) stimulated the pro‐inflammatory response in macrophages, osteogenic differentiation of BMSCs, and angiogenic activity of HUVECs. Bone precursor cells and endothelial cells contribute to form cartilaginous bony callus (soft callus), which bridge the gap between the bone fragments.^141^, ^142^ Chronic inflammation is deleterious to proceed to heal the wound trajectory. Therefore, immunomodulatory nanobiomaterials have been developed using RE nanoparticles such as ceria, which facilitate immunomodulatory action by switching M1 macrophage to M2. M2 macrophages recruit and activate precursor cells to form a cartilaginous soft callus. Soft callus, along with endothelial cells and osteoblasts, then progresses to hard callus formation, also known as primary bone formation; this stage represents the most active period of osteogenesis.^143^ Following these processes, the bone remodeling phase begins with coordinated osteoblast and osteoclast activities. Reabsorption of callus tissues by osteoclast is followed by lamellar bone formation. The ROS‐producing ability of RE nanoparticles such as ceria activates the RANKL pathway to induce osteoclastogenesis.^78^, ^79^ Moreover, angiogenesis is a critical factor for bone remodeling because it provides the appropriate conditions for osteoblast and osteoclast activities.^143^, ^144^, ^145^ The RE materials such as europium has the potent role of angiogenic activity via ROS production.^127^, ^128^

## 5 APPLICATION OF RE SMART NANO‐BIOMATERIALS IN BONE TISSUE ENGINEERING

Bio‐implants are orchestrated specialized materials that render the ability to replace or restore the specific functions of the damaged organs or tissues.^146^, ^147^ One of the recently identified such materials belongs to RE metal groups. The different RE nanomaterial synthesis methods and their physicochemical properties are listed in Table 1. In addition, RE nanomaterials have a lot of biological applications. Reports from literature had report antioxidants potential of ceria,^125^, ^126^ osteo‐angiogenic effects of europium,^127^, ^128^, ^129^ laser irradiation property of neodymium,^132^, ^133^ and contrast imaging potential of Gd.^130^, ^131^ Various biological applications, especially concerning bone tissue engineering application of RE materials, are summarized in Table 2. The outcomes of bone fracture healing strategies are still not satisfactory due to the lack of osteoinduction, osteoconduction, immunomodulation, and osteointegration ability of biomaterials. The use of emerging RE nanomaterials has the potential to address these challenges. In the past two decades, significant advancements have been made using RE materials in bone implants and prostheses design. This review attempts to comprehensively exemplify the potential usage of RE elements in bone graft and implant development. We profoundly discuss the challenges in using RE nanomaterials in bone regenerative medicine, particularly in the osteogenic process.

**TABLE 1:** RE nanoparticle synthesis and their properties

S.no	Method	Nanoparticles	Reductant/modification	Properties	References
1.	Hydrothermal	Cerium oxide (CeO_2_)	Sodium dodecyl sulfate	Weak agglomeration	148
2.	Hydrothermal	Pr‐, Gd‐, and Sm‐doped ceria nanoparticles	20% Pr and Sm 10% Gd	Weak agglomeration (13–25 nm)	149
3.	Solution casting	Ce_2_O_3_	PLGA	Sustained release of the ceria nanoparticles	150
4.	Flame spray pyrolysis	Nanoceria	Heparin and 3‐amino propyl tri‐ethoxy silane	12 nm	151
5.	Sol–gel	Ce_2_O_3_, Ga_2_O_3_ doped ZnO	0.2% Ce_2_O_3_ and 1.0% Ga_2_O_3_	Mesoporous	152
6.	Plasma spraying	CeO_2_	Calcium silicate	Antioxidant	153
7.	Plasma spraying	CeO_2_	Titanium	Antioxidant	154
8.	Sol–Gel	CeO_2_ nanoparticles	Oligochitosan alginate and gelatin	Injectable hydrogel	155
9.	Microemulsion	Ceria nanoparticles	Alendronate‐PEG 600	Endochondral ossification	125
10.	Melt quench and Polymer foam replication	Ce_2_O_3_ and Ga_2_o_3_	Borate (13‐93b3)	Bioactive glass powders	156
11.	Plasma sprayed	CeO_2_	Calcium silicate	Antimicrobial activity	157
12.	Ultrasonication	EuF_3_‐ and TbF_3_‐coated multiwalled carbon nanotubes	Sodium dodecyl sulfate	10 nm thickness of coating	158
13.	Solution synthesis	Eu^3+^‐doped Y_2_O_3_	Alumina nanoparticles	Ultrathin films	159
14.	Microemulsion	Eu (DBM)_3_ dibenzoylmethanate phenanthroline nanoparticles	Triton X‐100, Octanol, and cyclohexane	40 nm in size, spherical shape, and good dispersibility	160
15.	Chemical etching	Re_10_Pb_25_F_65_ Re‐Er^3+^, Yb^3+^, Eu^3+^, Dy^3+^, Ho^3+^, Tm^3+^	Oxyfluoride nano‐glass‐ceramics	8 nm diameter	10
16.	Solution Combustion‐fluoridation	RE‐doped Lu_2_O_3_ and Y_2_O_3_ powders	Eu^3+^‐doped and codoped with Yb^3+^/Ho^3+^	200–300 nm size	161
17.	Co‐precipitation‐solvothermal	Eu‐doped Y_2_O_3_	Aqueous and ethylene glycol	Y2O3:Eu wires and spherical, photoluminescence	162
18.	Conjugation	Eu^3+^‐doped Gd^3+^	Fe_3_O_4_ nanoparticles via a PEG‐NH2 linker	Water‐soluble cell fluorescence imaging	40
19.	Microwave	Tb^3+^‐doped Eu^3+^	Polyethyleneimine	12 nm multicolor luminescent LaF3	163
20.	Sol–gel	Eu^3+^‐, Sm^3+^‐, and Tb^3+^‐doped TiO_2_	Titanium (IV)‐isopropoxide, water, ethanol, and nitric acid in the molar ratio of 1:3:20:0.08	Red emission in Eu^3+^, Sm^3+^ doped TiO_2_	164
21.	Sol–gel	Eu(III)	Europium(III)‐doped yttrium, lanthanum, and gadolinium oxides	Sub‐10 nm, luminescent properties	165
22.	Emulsifier‐free emulsion polymerization	Eu nanoparticles	Oleic acid and sodium undecylenate modified Fe_3_O_4_	120 nm exhibit superparamagnetism	166
23.	Conjugation	Gd‐FITC mesoporous silica nanoparticles	Diethylene triamine pentaacetic acid, phenyl thiourea, tetraethyl orthosilicate, and cetyltrimethylammonium bromide	Green fluorescence and paramagnetism	167
24.	Thermolysis (>250°C)	Er^3+^/Yb^3+^ co‐doped NaGdF4	Oleic acid, 1‐octadecene, sodium trifluoroacetate, polyacrylic acid, and chloroform RGD	32 ± 9 nm in size, optical, and magnetic properties	168
25.	Green chemistry	Gd nanoparticles	Dextran, ammonium hydroxide	Ultrafine sub‐10 nm	169
26.	Molecular dynamics simulations	Metallofullerenol Gd@ C_82_(OH)_22_	Fullerene C82	Inhibition of MMP‐2 and MMP‐9	170
27.	Solvothermal	GdPO_4_•H_2_O nano‐bundles	NH_4_H_2_PO_4_, HA, and PLGA	~1 μm in length, ~30 nm in width, paramagnetism	171
28.	Polyol	Gadolinium (III) oxide	3‐glycidyloxypropyl trimethoxysilane, Bisphosphonate	70 nm, and long‐term follow‐up imaging studies	131
29.	Lyophilization method	GdPO_4_/CTS	Chitosan	Porous scaffolds	172
30.	Lyophilization method	Gd‐doped MCS/CTS (Gd‐MCS/CTS) scaffolds	CTAB, NH_3_·H_2_O, TEOS	Hierarchically porous structures	173
31.	Thermolysis (>250°C)	Er^3+^/Yb^3+^ co‐doped NaGdF_4_	Oleic acid, 1‐octadecene, sodium trifluoroacetate, polyacrylic acid, chloroform RGD	32 ± 9 nm in size, optical, and magnetic properties	168
32.	Green chemistry	Gd nanoparticles	Dextran, ammonium hydroxide	Ultrafine sub‐10 nm	169
33.	Hydrothermal	Neodymium oxide	Acetic acid	Fibrous/rod‐like particle	174
34.	Solvothermal	Neodymium oxide	Nitric acid/acetic acid	Fibrous/needle‐like particle	175
35.	Chemical	Nd(OH)_3_	Borohydride	30–100 nm	176
36.	Microemulsion	Nd(OH)_3_	n‐butanol, n‐octane, CTAB	Cube, sphere, and oval like	177
37.	Radiofrequency sputtering	Nd‐doped TiO_2_	TiO_2_ and metallic Nd (RF:13.56 MHz)	Red luminescence	178
38.	Wet co‐precipitation	NdPO_4_	NH_4_H_2_PO_4_	92 nm, monoclinic, spherical	179
39.	Inverse microemulsion and sol gel	Neodymium oxalate	Organically modified silane (Ormosil)	10–40 nm, violet emission	180
40.	Polyol	Neodymium oxide	Diethylene glycol, NaOH	2–5 nm in size spherical shape	181
41.	Sol–gel	CeO_2_, Pr_2_O_3_, and Nd_2_O_3_	Citric acid	10–30 nm, spherical shape	182
42.	Chemical reduction	Nd	Sodium borohydride, hydrazine hydride, ammonia	Spherical, cube, and rod	18
43.	Electrospinning	Nanofiber	Polyvinyl acetate	Crystalline 20 nm diameter	183
44.	Sol–gel	Pr^3+^	Citric acid, ammonia solution	Spinel cubic crystal and larger ionic radii	184
45.	Polyol	Pr_6_O_11_	Diethylene glycol and sodium hydroxide	10 nm	185
46.	Hydrothermal	Ce/Pr‐CQDS	EDTA, Glycine	Hydroxyl radical scavenging	186
47.	Ball milling	SmCo_5_ and PrCo_5_	Dry HEBM under argon, Wet HEBM–heptane, and oleic acid	10 nm	187
48.	Surface functionalization	Sm‐doped YVo_4_	Citrate and polyvinyl pyrrolidine	20–50 nm	188
49.	Emulsion	Sm153	EDTMP	200–500 nm	189
50.	Thermal decomposition	Y_2_O_3_ nanoparticles	Oleic acid	30 nm, green fluorescence at room temperature	190
51.	Microwave irradiation method	Terbium hydroxide nanorods	NH_4_OH	340 nm length, 65 nm width	191
52.	Solvothermal	YbFeO_3_(o‐YbFeO_3_)	Ytterbium acetate, Yb chlorides, and iron acetylacetonate	Hexagonal orthorhombic perovskite structure	192

**TABLE 2:** Applications of RE smart nano‐bio materials in bone tissue engineering

S.No	RE materials	Biological property	Model	Mechanism/pathway	References
1.	Nanoceria	Antioxidants	Homozygous tubby (tub/tub) mice	Neuroprotection genes	193
2.	CeO_2_ nanoparticles	Antioxidant	Osteoblastic cell line (MC3T3‐E1)	ROS production	153
3.	CeO_2_ nanoparticles	Antioxidant	MC3T3‐E1	Wnt/β‐catenin	194
4.	CeO_2_ nanoparticles	Antioxidant	MC3T3‐E1	Osteoradionecrosis	195
5.	Cerium (III)	Osteoclastogenesis	RAW264.7	NADPH oxidase 1	79
6.	CeO_2_ nanoparticles	Pro‐angiogenic property	MSCs	Increased Ca^2+^ level, HIF‐1α, VEGF signaling	196
7.	SmCeO_2_	Pro‐angiogenic property	Endothelial cells	p38MAPK/HIF‐1α	197
8.	CeO_2_ nanoparticles	Osteoinductive and anti‐inflammatory	BMSCs, RAW264.7	BMP2 and TGF‐β1, CD206, IL‐1ra, and IL‐10	194
9.	Ce^3+^	Osteoinductive and anti‐inflammatory	BMSCs	Smad/BMP	198, 199
10.	Ceria nanoparticles	Endochondral ossification	Mice critical‐sized bone defects	DEAH (Asp‐Glu‐Ala‐His) box helicase 15 and p38 MAPK	125
11.	Nanoceria	Anti‐angiogenic and pro‐inflammatory	Vldlr null mice	ERK 1/2, JNK, p38 MAP kinase, and Akt	200
12.	Oligochitosan coated CeO_2_ nanoparticles	Anti‐angiogenic and pro‐inflammatory	Human retinal pigment epithelium‐19 and umbilical endothelium cell lines	Inhibition of VEGF and inflammatory‐related protein expression	155
13.	Ce (III)‐based alginate/hyaluronate hydrogel	Osteoconductivity and antimicrobial ability	MG63 cells, *Staphylococcus epidermidis*, *Pseudomonas aeruginosa*, and *Candida albicans*	MG63 cell viability	201
14.	Ceria inclusion in the graphene hydroxyapatite (GR‐HA) matrix	Osteoconductivity and antimicrobial ability	MG63 cells, *Staphylococcus aureus*, *Staphylococcus epidermidis*, *Pseudomonas aeruginosa*	Expression of the osteoblastic genes Runx2, Col 1, ALP, BMP‐2, OC and OPG	202
15.	Ceria and silver‐reinforced HA composite	Antioxidant and antibacterial	*E. coli* and *S. aureus*	Mechanical integrity and cytocompatible	126
16.	CeO_2_ incorporated calcium silicate	Dental implants and antimicrobial activity	*E. faecalis*	ALP, OCN, and BSP	157
17.	Ceria nanoparticles on the poly‐l‐lactide scaffold	Cell‐material interactions	Human MSCs and osteoblast‐like cells (MG63)	Ce^4+^ enhances proliferation, migration, and adhesion behavior	14
18.	Cerium	Osteogenic differentiation and mineralization	MC3T3‐E1	Runx2, BMP2, ALP, BSP, Col I, and OCN	77
19.	Nanoceria	Osteogenic differentiation	BMSCs	Dose‐dependent manner, 24–72 h	203
20.	Ceria	Osteogenic differentiation	MSCs	TGF‐β/BMP	204
21.	Ceria	Osteogenic differentiation	BMSCs	Smad/BMP	205
22.	Ceria‐stabilized zirconia/alumina	Mandibular implant	Clinical report	Elasticity equivalent to that of a cobalt‐chromium	206
23.	Cerium‐based zirconia/alumina composite	Osteogenic response	MC3T3‐E1 and male Sprague–Dawley rats	Osteogenic response in vitro and the osseointegration capability in vivo	207
24.	Nano CeO_2_	Bone regeneration	BMSCs and male Sprague–Dawley rats	Enhancing bone regeneration in a critical‐size defect rat model	208
25.	CeO_2_ nanoparticles‐modified bioglass scaffolds	Osteogenic differentiation	Human BMSCs and in vivo rat, cranial defect models	ERK pathway, collagen deposition, osteoclast formation, and bone regeneration	76
26.	Nanocrystalline CeO_2_	Dentinogenesis	Chinchilla breed rabbits	Dentin and bone regeneration effectively	209
27.	CeO_2_ nanoparticles	Chemotherapeutic action	Osteosarcoma cell line SAOS‐2	pH‐sensitive manner	210
28.	CeO_2_ nanoparticles	Osteoclastogenesis	Bone marrow‐derived macrophages	ROS‐mediated RANKL pathway	78
29.	Eu(III) complex	Contrast agent	Bovine tibia specimens	Bone structure analysis	211
30.	Gold nanoparticles conjugated with the europium	Luminescent probe	Human platelets	Targeted the platelets in low pH 6.5	212
31.	Europium hydroxide nanoparticles	Angiogenesis	Endothelial cells	PI3K/Akt	213
32.	Europium (III) hydroxide	Pro‐angiogenic properties	Endothelial cells	MAPK pathway	129
33.	Gd_2_O_3_:Eu^3+^ nanotubes	Bone mineral density	MC3T3‐E1	High ALP activity, mineralization, BMP signaling pathway	214
34.	Bioactive glass incorporated europium scaffolds	Luminescent property and new bone formation	Osteoporotic bone defects in OVX rats	Bone formation	128
35.	Europium‐doped mesoporous silica nanospheres	Pro‐inflammatory and osteogenic differentiation	Macrophage and HUVECs	New bone formation at a critical‐sized cranial defect site	215
36.	Europium‐doped bioactive glass nanoparticles	Osteogenic differentiation	Human MSCs	ALP activity, COL I secretion, ALP, Col I, OPN, Runx2	127
37.	Eu^3+^‐doped nanohydroxyapatite	Luminescent property and osteogenic differentiation	hASCs	GSK3β /β‐catenin	216
38.	Gd doped FITC silica nanoparticles	Differentiation into adipocytes, osteocytes, and chondrocytes	Human MSCs	Green fluorescence and paramagnetism	167
39.	RGD functionalized Er^3+^/Yb^3+^ co‐doped NaGdF_4_	Tumor angiogenesis	U87MG tumor cells	Target the α_v_β_3_ integrin–expressing tumor cells	168
40.	Gd@C_82_(OH)_22_	High antitumoral efficacy	Molecular dynamics simulations	Inhibit MMP‐2 activity	170
41.	Gd‐based nanoparticles	Tumor angiogenesis	Balb/c tumor‐bearing mice	Determination of tumor boundary by MR imaging	169
42.	GdPO_4_H_2_O nanobundles	MRI and X‐ray tracing and osteogenesis	MC3T3‐E1 and in vivo rabbit radius defects	OCN and mineralization	171
43.	GdPO_4_/CTS scaffolds	Osteoconductivity	Rabbit BMSCs	ALP, Runx‐2, OCN, Col‐I, and Smad/Runx2	172
44.	Gd‐doped MCS/CTS	Osteogenic differentiation	Rabbit BMSCs	Wnt/β‐catenin	173
45.	Gd‐BG scaffolds	Osteogenic differentiation	Human BMSCs	Akt/GSK3β	217
46.	Ca−P‐coated Mg−Zn−Gd scaffolds	Orthotopic reconstruction of large‐sized orbital bone defect healing	Canines	CGRP‐mediated angiogenesis and osteogenesis	101
47.	Gadolinium MRI enhancer	Assessment of perfusion in carpal bones	Kienbock's disease	Diagnose altered perfusion in patients with Kienbock's disease	218
48.	Gadolinium (III) oxide nanoparticles	Monitor in vivo implantation	Condyle defect rat model	Long‐term follow‐up imaging studies	131
49.	Gadolinium (III) nanocages	MRI imaging	KPC transgenic mouse models	Detect neuropilin‐1‐positive in pancreatic cancer	219
50.	Gadolinium	Whole‐body magnetic resonance imaging	Breast cancer, prostate cancer, and lung cancer patients	Detection of bone metastasis	130
51.	Yb^3+^/Ho^3^ Co‐doped apatite nanoparticles	Bone regeneration	MG63 cells and New Zealand white rabbits	Distinguish implanted material from bone tissue	220
52.	Magnetic lanthanum‐doped HA/CS scaffolds	Macrophage polarization and bone regeneration	Rat bone marrow mesenchymal stem cells	Upregulation of Smad 1/5/9 pathway	92
53.	Lanthanum phosphate chitosan scaffolds	Osteogenic differentiation	BMSCs and rat critical‐sized calvarial defect sites	Wnt/β‐catenin signaling pathway	221
54.	La^3+^ ions calcium silicate chitosan bone scaffolds	Osteogenic differentiation	Rabbit BMSCs	TGF signal pathway	222
55.	Nd: YVO_4_	Laser oscillator for drill the cortical bone	Femoral bone of a pig	160 mW for 0.75‐mm thick drilling	223
56.	Nd: YAG silicon carbide on Ti6Al4V	Laser irradiation on bone healing	Osteoblast	Bone healing	224
57.	High‐power, low‐level Nd: YAG laser	Laser irradiation on bone healing	MC3T3‐E1 osteoblasts	BMP‐2‐related signaling pathway	132
58.	Nd:YAG laser with EMP	Healing intrabony defects	Periodontal disease	Probing depth decrease and increased clinical attachment level (CAL)	225
59.	Nd: YAG laser with SRP	Periodontal inflammatory response	Periodontal inflammation	Plaque index (PI), gingival index (GI), probing pocket depth (PPD), and marginal bone loss	226
60.	Nd_2_O_3_	Inflammatory response	Human bronchial epithelial cells	STAT3	227
61.	Nd nanoparticles	Redox‐mediated angiogenic response	EA.hy926 endothelial cells	PKM2‐NOX4 signaling pathways	18
62.	Nd:YAG	Laser irradiation	Male Wistar rat	Accelerate bone metabolism during tooth movement	228
63.	Nd:YAG Q‐switch laser	Antimicrobial	Peri‐implantitis	Disinfected the contaminated implant	229
64.	Nd‐Ca‐Si silicate glasses and alginate composite hydrogels	Anticancer and wound healing bioactivity	HUVEC cells, nude mice, and BALB/c mice	Thermal therapy for cancer treatment and burn wound healing	230
65.	Samarium with EDTMP and Technetium‐99m	Targeted delivery for bone metastasis	Male Wistar rats	150 min accumulation and release of EDTMP at bone tissue	189
66.	Sm^3+^‐doped P_2_O_5_ glass‐reinforced hydroxyapatite	Osteogenesis and antimicrobial	MG63 cells, *Staphylococcus aureus*, *Staphylococcus epidermidis*, and *Pseudomonas aeruginosa*	F‐actin cytoskeleton organization and cell proliferation in MG63 and potent antimicrobial activity	231
67.	Y_2_O_3_ nanoparticles incorporated polycaprolactone scaffolds	Cell proliferation and angiogenesis	Fibroblasts (L‐929) and osteoblast‐like cells (UMR‐106)	VEGF and EGFR	232
68.	Er:YAG laser irradiation	Evaluate the moisture content, roughness, and thickness	Cortical bone	Optical coherence tomography (OCT)	233

Abbreviations: ALP, alkaline phosphatase; BMSCs; bone marrow mesenchymal stem cells; BSP, bone sialoprotein; CGRP, calcitonin gene‐related polypeptide‐α; EDTMP, ethylenediamine tetramethylene phosphonic acid; MSCs, mesenchymal stem cells; OCN, osteocalcin; VEGF, vascular endothelial growth factor.

### 5.1 Cerium

Cerium is the most abundant RE element, approximately 50–60 ppm found on the earth's surface. Cerium exhibits unique redox behavior due to its electron configuration, filling the 4f orbital in the ground state and standard oxidation numbers of +3 or + 4. Oxide forms of cerium include cerium oxide or ceria (CeO_2_), and dicerium trioxide or sesquioxide (Ce_2_O_3)_ has been broadly utilized for various applications, such as electrolytes in fuel and solar cells, detection systems, surface polishing, and catalysis. The redox equilibrium between two oxidation states results in the ROS and reactive nitrogen species (RNS) regulation. At the nanoscale level, the reactivity of CeO_2_ is more effective as the high surface‐to‐volume ratio results in elevated surface oxygen vacancies, which is responsible for the enhanced biological activities such as antimicrobial, antioxidants, and angiogenic responses.^91^ The applications of CeO_2,_ especially in bone formation, are discussed in the following sections.

#### 5.1.1 Redox modulator

Redox signaling is essential for physiological and pathological conditions. Under physiological conditions, there will be a balance between oxidants and antioxidants, which maintains the redox state at the threshold level. The redox states altered beyond the tolerable threshold level lead to apoptosis. Oxidative stress caused by generating abundant ROS in the living system is obnoxious. The body itself has a defense mechanism to modulate such redox states, whereas, in some pathological conditions like bone fracture microenvironment, the levels of ROS are abundantly high and affect bone reconstruction. Excessive ROS production can induce osteoclastogenesis and suppresses the osteoblastic differentiation process. Therefore, it is essential to balance the equilibrium by using antioxidants to modulate the redox states. Nanoceria acts as an antioxidant therapeutic. The different sizes (5, 15, 30, or 55 nm) of ceria particles biodistribution had been analyzed by intravenous injection in rats.^28^ The nanoceria was detected in blood, brain, liver, and spleen. The liver and spleen contain a large percentage of the injected dose, with no significant clearance over 720 h and very little nanoceria entered brain parenchyma. Superoxide dismutase mimetic activity retains in PLGA encapsulated ceria nanoparticles for 90 days under different pH.^150^ Plasma‐sprayed CeO_2_ coating enhances superoxide dismutase activity and reduces ROS in hydrogen peroxide (H_2_O_2_)‐treated osteoblasts.^153^ The heparin‐functionalized nanoceria enhances cellular uptake and ROS scavenging.^151^

**FIGURE 1:**
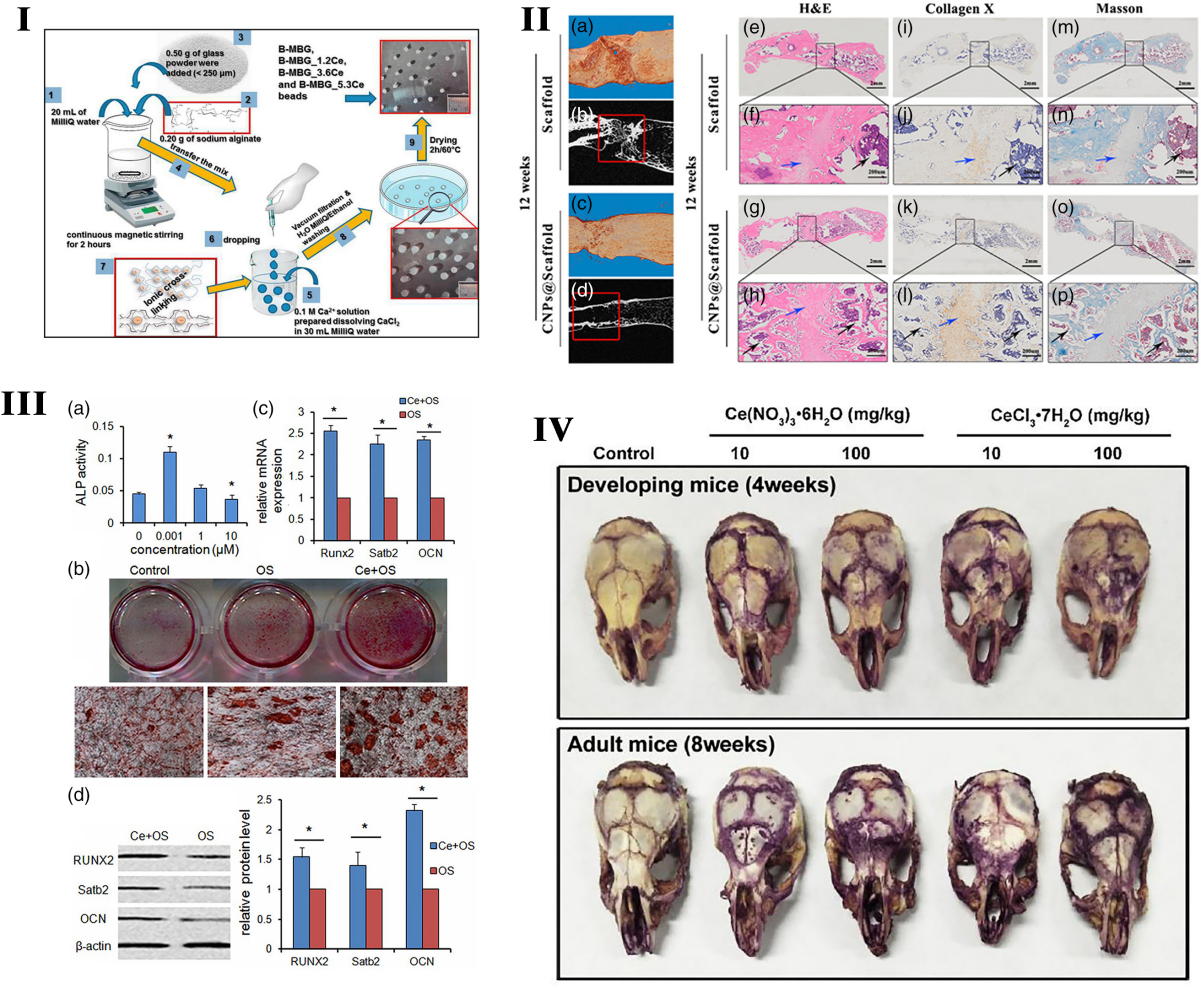
I. Schematic representation of the preparation of the alginate/glass beads with ceria to prevent oxidative stress in MC3T3‐E1. *Source*: Reprinted with permission from ref. 234. Copyright 2019, Elsevier. II. The effect of cerium‐doped nanoparticles on osteogenesis (a–d). Representative micro‐CT (b, d) and 3D reconstruction (a, c) images of femurs 12 weeks after ceria‐based scaffold implantation. The red solid line frame outlines the bone defect area. (e–h) H&E staining at 12 weeks post‐surgery. (i–l) Collagen X IHC staining at 12 weeks post‐surgery. (m–p) Masson's trichrome staining at 12 weeks post‐surgery. The solid black box represents the enlarged defect area. Blue arrowheads indicate hypertrophic chondrocytes, and black arrowheads represent new trabecular bone formed by endochondral ossification (*n* = 3/group). *Source*: Reprinted with permission from ref. 125. Copyright 2019, John Wiley & Sons, Inc. III Ce promotes bone marrow mesenchymal stem cells (BMSCs) osteogenic differentiation ex vivo. (a) BMSCs were treated with various concentrations of Ce (0, 0.001, 1, 10 μM) for 7 days and assessed by measuring the alkaline phosphatase (ALP) activity. (b) BMSCs were treated with standard, OS, and OS + Ce medium for 21 days and assessed by alizarin red S staining. (c) Quantitative real time PCR analysis indicated that the mRNA expressions of Runx2, Satb2, and OCN were significantly up‐regulated in the BMSCs treated with Ce (0.001 μM) for 7 days compared to the control group. (d) Western bolt analysis showed the expressions of RUNX2, Satb2, and OCN proteins were up‐regulated after treatment with Ce (0.001 μM) for 7 days. Data are presented as mean ± *SD* from a representative of three separate experiments. *p < 0.05. *Source*: Reprinted with permission from ref. 205. IJCEP Copyright 2014. (IV) The TRAP staining of mice skull treated with cerium for 9 days. *Source*: Reprinted with permission from ref. 79. Copyright 2019, Elsevier. TRAP, tartare resistant acid phosphatase

Radiation causes bone damage, including a decrease in osteocyte number and osteoblastic activity. CeO_2_ nanoparticles exhibit protective effects on irradiation‐induced osteoradionecrosis in MC3T3‐E1 cells by reducing oxidative stress.^195^ Further, increasing the content of CeO_2_ in HA coatings diminishes the H_2_O_2_‐induced inhibition of osteogenic differentiation and increases alkaline phosphatase (ALP) activity, calcium deposition activity, and mRNA expression levels of osteogenesis markers runt‐related transcription factor‐2 (RUNX2), ALP, and osteocalcin (OCN) in bone marrow mesenchymal stem cells (BMSCs). Furthermore, CeO_2_ induces the gene and protein expressions of β‐catenin and cyclin D1.^194^ Similarly, Varini et al. found that mesoporous glasses with 1.2% and 3.6% CeO_2_ prevent oxidative stress improves MC3T3‐E1 cell proliferation.^234^ The schematic representation of the preparation of the alginate/glass beads with ceria is given in Figure 1I. The topical application of water‐soluble CeO_2_ nanoparticles (nanoceria) accelerates the healing of full‐thickness dermal wounds in mice by reducing oxidative damage to cellular membranes. Furthermore, nanoceria enhances the proliferation and migration of fibroblasts, keratinocytes, and vascular endothelial cells.^235^

The imbalance in the microenvironmental conditions such as changes in pH, necrotic cells, and invasion of microorganisms elevates the ROS levels in bone fracture environments and osteoporotic conditions.^236^, ^237^, ^238^ Elevated ROS levels hinder the recruitment of osteoblast precursors and delay the healing process. The H_2_O_2_ level above 0.3 mmol modulates oxidative stress and inhibits the osteogenic differentiation of odontoblastic cells and preosteoblastic MC3T3‐E1 cells via ERK and NFkB pathways.^239^ In contrast, the odontoblasts cells treated with H_2_O_2_ at concentrations below 0.3 mmol/L display a significant increase in ALP activity and matrix mineralization. Another study demonstrated that H_2_O_2_‐induced oxidative stress enhances differentiation of calcifying vascular cells and inhibits differentiation of bone cells, which causes either atherosclerosis by the accumulation of lipids in the vessel wall or osteoporosis by lack of osteoblast mineralization^236^ Even though nanoceria acts as an antioxidant, nanoceria also mimics the activity of superoxide dismutase,^240^, ^241^ catalase and nitric oxide synthase^242^ maintaining some basal level ROS and redox states, which are mainly dependent on catalytic activity and oxidation potential such Ce3+ and Ce4+.^14^, ^199^ The catalytic properties and biomedical applications of cerium oxide nanoparticles were critically reviewed by Walkey et al., the interested readers can be read it for further information.^243^ The microenvironmental conditions played a significant role in the production of ROS. Acidic environments like cancer, ceria nanoparticles favor the scavenging of superoxide radical over the hydroxyl peroxide resulting in accumulation of the ROS, which can be used for sensitization of cancer cells.^244^ Zhou et al. claimed that elevation of intracellular ROS level by cerium (III) enhances the expression and activity of NADPH oxidase 1, which further activates the RANKL‐dependent osteoclasts differentiation, and the cerium (III) activated osteoclasts exhibit higher bone resorption activity.^79^ The Figure 1IV depicted the osteoclastogenic effect of cerium by tartare resistant acid phosphatase (TRAP) staining. Another study reported that CeO_2_ nanoparticles facilitated osteoclast formation at lower concentrations via the RANKL pathway. A higher concentration of CeO_2_ inhibited osteoclastogenesis by inducing apoptosis in bone marrow‐derived macrophages by modulating cellular ROS levels.^78^ Recent research attempts with poly(1,8 octanediol‐co‐citrate), beta‐tricalcium phosphate, and CeO_2_ nanoparticles had developed the porous, biocompatible, bioactive, and free‐radical scavenging RE nanomaterials.^245^ The Ce_6_ upconversion nanoparticles act as photosensitizers that excite at 808 nm and convert NIR to visible photon energy. This event generates toxic ROS in cancer cells through the Fenton‐like reaction by Fe(OH)_3_ compound and enhances the tumor treatment efficacy.^246^

#### 5.1.2 Angiogenesis and immunomodulation

Insufficient blood vessel formation is a critical problem that hampers the clinical application of bone grafts. The scaffolds modified with CeO_2_ nanoparticles improve the proliferation and inhibit the apoptosis of MSCs. Meanwhile, it activates the calcium channel enhancing intracellular free Ca^2+^ level in MSCs, which subsequently augments the stability of hypoxia‐inducible factor‐1 alpha (HIF‐1α) and VEGF expression. The improved paracrine signaling of VEGF promotes the proliferation, differentiation, and tube formation ability of endothelial progenitor cells and significantly improves the blood vessel distribution inside of bone scaffolds.^196^ Physicochemical properties like Ce^3+^/Ce^4+^ ratio, surface charge, size, and shape of cerium nanoparticles influence the angiogenesis process. The Ce^3+^/Ce^4^ ratio modulates the intracellular oxygen environment by stabilizing HIF‐1α endogenously and promotes angiogenesis.^247^ Mesoporous sol–gel glasses substituted with Ce_2_O_3_, Ga_2_O_3_ (both 0.2% and 1.0%), and ZnO (0.4% or 2.0%), contain well‐interconnected ultra‐large pores (pores >400 μm) ideal for vascular ingrowth and proliferation of endothelial cells.^152^ The functional nanoconjugates of SmCeO_2_ trigger endothelial cell proliferation and induce the growth of blood vessels in the chick embryo. The enhanced expression of pro‐angiogenic markers (p38MAPK/HIF‐1α) by these functional nanoconjugates might be the plausible signaling mechanism of the pro‐angiogenic property.^197^ Endochondral bone regeneration is similar to long bone defect healing, which needs angiogenesis and osteogenesis. The micro emulsion‐based alendronate‐anchored polyethylene glycol‐modified ceria nanoparticles (CNPs) accelerated vascular invasion. They enhanced endochondral ossification‐based bone regeneration by activating RNA helicase, DEAH (Asp‐Glu‐Ala‐His) box helicase 15 (DHX15). CNPs enhance the proliferation and hypertrophic differentiation of BMSCs by stimulating the DHX15–p38 MAPK axis. Further inhibition of DHX15 by shRNA affected the expression of hypertrophic genes Runx2, MMP13, and Col10α1, which confirmed the importance of DHX15 in hypertrophic differentiation of BMSCs.^125^ The effect of cerium‐doped nanoparticles on osteogenesis is shown in Figure 1II,III.

The aberrant angiogenesis causes lethal effects in some neurodegenerative conditions and cancer metastasis. Nanoceria inhibits the expression of genes associated with inflammation and angiogenesis in the retina of Vldlr null mice representing a novel therapeutic strategy to treat age‐related macular degeneration (AMD) and other neurodegenerative diseases. Nanoceria causes inhibition of pro‐inflammatory cytokines and pro‐angiogenic growth factors and upregulation of several cytokines and anti‐angiogenic genes in the Vldlr^_/_^ retina. Nanoceria inhibits the activation of ERK1/2, JNK, p38 MAP kinase, and Akt.^200^ Similarly, the water‐soluble oligochitosan‐coated CeO_2_ nanoparticle‐loaded injectable hydrogel shows biocompatibility and radical‐scavenging effect.^155^ Furthermore, it downregulates the expression of angiogenic proteins and pro‐inflammatory cytokines in AMD cellular models like human retinal pigment epithelium‐19 and umbilical endothelium cell lines.^155^ It also has been documented that nanoceria alleviates the endometrial lesions induced in the mice model by decreasing oxidative stress and inhibiting angiogenesis.

Moreover, nanoceria was also observed to protect endometriosis‐related adverse effects on the oocytes, which is critical for a successful pregnancy.^248^ The genotoxicity studies in liver cells revealed that the high dose (1000 mg/kg body weight) of ceria nanoparticles induces DNA damage in peripheral blood leukocytes, micronucleus formation in blood cells, and total cytogenetic changes in the bone marrow. Ceria nanoparticles exhibit higher tissue distribution and greater clearance in large fractions through urine and feces than CeO_2_ bulk, whereas the maximum amount of micro‐sized CeO_2_ excretes in feces.^249^ Nanoceria significantly inhibits the production of ROS in A2780 ovarian cancer cells. Nanoceria treatment also inhibits VEGF165‐induced proliferation, capillary tube formation, activation of VEGFR2 and MMP2 in HUVECs. Thus, nanoceria can be used as an anti‐angiogenic therapeutic agent during cancer treatment.^250^ This pro‐angiogenic and anti‐angiogenic potential of ceria‐based nanoparticles might be related to the dose of ceria content in the nanoparticles, the cell type, and disease condition. Optimizing the proper dose of cerium in the ceria nanoparticles is crucial for pro‐angiogenic effect‐mediated bone defect healing.

Plasma spraying technique‐based CeO_2_‐coated (CS‐10Ce and CS‐30Ce) calcium silicate materials have shown good osteogenic responses in bone marrow‐derived MSCs (BMSCs) by increasing the expression of osteoinductive molecules BMP2 and TGF‐β1. This effect limits inflammatory reactions by up‐regulating the expressions of anti‐inflammatory M2 macrophage markers (CD206, IL‐1ra, and IL‐10) in RAW264.7 macrophages.^194^ Ce^4+^/Ce^3+^ (i.e., 0.46, 1.23, and 3.23) ratios of CeO_2_ nanoparticles applied to titanium substrate surfaces by magnetron sputtering elevate the M2 macrophage polarization and anti‐inflammatory cytokine secretion resulting in new bone formation and osseointegration.^199^ Since immunomodulation plays a vital role in bone defect healing and implant success, the immunomodulatory potential of nanoceria could be applied in bone tissue engineering and implantology. Similarly, the T cells, B cells, neutrophils, and other immune cells participate in the bone regeneration cascade.^251^, ^252^ The effect of RE metal, including cerium‐based nanomaterials, on the activation and expansion of the T cells, B cells, neutrophils, and other immune cells during bone defect healing is still unknown.^253^

#### 5.1.3 Antimicrobial activity

Due to the antioxidant property of ceria, it has been widely used as an antimicrobial agent. Alginate/hyaluronate and Ce (III) ions based hydrogel shows bioactive and antimicrobial ability against *Staphylococcus aureus*, *Staphylococcus epidermidis*, *Pseudomonas aeruginosa*, and *Candida albicans* without compromising the osteoconductivity. The antimicrobial ability of Ce(III) is observed in Ce^3+^ ion incorporated hydrogel. A higher Ce(III) concentration in the hydrogel leads to an even stronger antimicrobial activity. The Ce^3+^ in cerium oxide is the key component of antioxidant activity to overcome free‐radical formation during the cellular growth process. Further, nanoceria decreases NO production in macrophages and in tissues of C57BLK6 mice for alleviating the pro‐inflammatory response caused by the infectious agents, which could be the mechanism of ROS scavenging ability of nanoceria‐mediated anti‐inflammation that serves as a treatment for a broad spectrum of inflammatory diseases.^201^, ^254^ The same research group also reported that ceria inclusion in the graphene hydroxyapatite (GR‐HA) matrix induces antimicrobial resistance against *S. aureus*, *S. epidermidis*, and *P. aeruginosa* of the composite.^202^ Antioxidant ceria and antibacterial silver reinforce HA composite with enhanced mechanical and cytocompatible properties and show antibacterial efficacy of ~61% for *Escherichia coli*and ~53% for *S. aureus*.^126^ Plasma‐sprayed CeO_2_‐incorporated calcium silicate coating in dental implants shows better biocompatibility, upregulates mRNA expression levels of ALP, OCN, and bone sialoprotein (BSP), and intensifies antimicrobial activity against *Enterococcus faecalis* ^157^

#### 5.1.4 Osteogenesis

Unique biological properties of ceria nanoparticles such as antioxidants, anti‐inflammatory, pro‐angiogenic, and antimicrobial nature suggest ceria as an appropriate biomaterial for bone tissue engineering applications. The ceria nanoparticles on the poly‐l‐lactide scaffold surface promote hMSCs and osteoblast proliferation, migration, and adhesion.^14^ The antioxidant properties of the CeO_2_‐incorporated HA coatings maintained intracellular SOD activity, reduced oxidative injure, and enhanced the osteogenic differentiation of BMSCs, probably through Wnt/β‐catenin signaling.^255^ The cerium ions influence the formation and structure of HA, as indicated by the apatite structure maintained by Ce^3+^ ions.^256^ The cerium has shown dose‐dependent osteogenic effects on MC3T3‐E1 cells. Cerium at concentrations of 0.0001, 0.001, 0.01, 0.1, or 1 μM promotes the proliferation and osteogenic differentiation of MC3T3‐E1 cells, as displayed by the upregulation of RUNX2 and BMP2 ALP, BSP, collagen I (COLI), and OCN. Whereas 1000 μM ceria inhibits osteogenic differentiation.^77^ Similarly, exposure to 1% ceria reduces ALP activity in MC3T3‐E1 cells, and cerium trichloride (CeCl_3_) stimulates MC3T3‐E1 cell proliferation.^203^ These results from the literature indicate that loading the proper dose of ceria in biomaterials is crucial for effective bone regeneration. The plasma‐sprayed CeO_2_^−^ coating with higher Ce^4+^ concentration elicits more significant effects than the CeO_2_ coating with Ce^3+^ concentration. The osteogenic differentiation is activated by RUNX2 expression and enhanced through increased ALP and OCN expression in BMSCs through the Smad‐dependent BMP signaling pathway.^198^ The nanoceria‐mediated osteogenic differentiation of BMSCs is dose‐dependent between 24 and 72 h. Prolonged incubation with nanoceria, that is, 14 days, inhibits the osteogenic differentiation. In contrast, nanoceria inhibits the adipogenic differentiation of BMSCs on Day 17, which conferred that the biomaterial doped with ceria should not give prolonged release and should be optimized for a better bone regenerative effect.^257^ Melt quench technique‐based bioactive borate (13‐93B3) glass powders containing up to 5 wt% Ce_2_O_3_ and Ga_2_O_3_ increases chemical durability, exhibits a good in vitro bioactive response, and has high in vitro HA forming ability making them promising candidates for bone tissue engineering applications.^156^ Ceria promotes osteogenic differentiation in MSCs by interacting with BMP receptors and activates TGF‐β/BMP signaling pathway by upregulation of RUNX2, which further up‐regulates osteoblast marker genes COLI and BMP2 at early stages, ALP, and OCN at later stages of differentiation further inhibits the adipogenic differentiation of MSCs by downregulation of an adipocyte marker PPARγ2.^204^

Smad‐dependent BMP signaling plays a vital role in the migration and osteogenic differentiation of BMSCs. Ceria promotes the phosphorylation of Smad1/5/8 and translocating to the nucleus via increased BMP2 expression. The activity of p‐Smad1/5/8 increases stromal cell‐derived factor‐1 (SDF‐1) and RUNX2 expression levels in BMSCs.^205^ The foamed ceria made up of CeO_2,_ and bovine hydroxyapatite (BHA) composites show potential free‐radical scavenging ability for developing orthopedic biomaterial.^258^, ^259^ Ceria‐stabilized zirconia/alumina nanocomposite exhibits an elastic and flexible property equivalent to a cobalt‐chromium alloy used as a mandibular implant.^206^ Intramuscular injections of CeO_2_ enhance muscle mass, glycogen, ATP content, and type I fiber ratio, resulting in higher muscle endurance.^260^ The cerium/zirconia/alumina composite enhances the osteogenic response in vitro and in vivo.^207^ Nano CeO_2_‐containing calcium sulfate hemihydrate composite with 5% w/w shows a higher bone regenerative potential.^208^ Freeze‐dried CeO_2_ nanoparticles‐modified bioglass scaffolds rapidly promote the proliferation and osteogenic differentiation of human BMSCs. The enhanced osteoinductivity of ceria‐bioglass scaffolds is mainly related to the activated ERK pathway. Rat cranial defect model revealed that ceria‐bioglass scaffolds accelerate collagen deposition, osteoclast formation, and bone regeneration compared to bioglass scaffolds.^76^ Nanocrystalline CeO_2_ promotes dentinogenesis in the damaged teeth root.^209^ All aforementioned osteogenic properties of cerium‐doped innovative nanomaterials indicate the potential applications of cerium in bone tissue engineering and implantology.

### 5.2 Europium

Europium is the least dense, the softest, and the most volatile member of the lanthanide series. The europium element was discovered in 1901 by French chemist Eugène‐Anatole Demarçay and was named for Europe. Europium occurs in minute amounts in many RE minerals such as monazite and bastnasite. The primary use of europium is in optical displays, TV screens, and fluorescent lamps. Europium is also used in scintillators for X‐ray tomography and as a source of blue color in light‐emitting diodes.^261^ The bio labeling property of europium ions has been used to synthesize the cyclen‐based europium (III) complex as a lanthanide luminescent contrast agent for bone structure analysis by incorporating the iminodiacetate functionalities as selective Ca(II) binding motifs. This contrast agent selectively visualizes the damaged bone structure (microcracks).^211^

**FIGURE 2:**
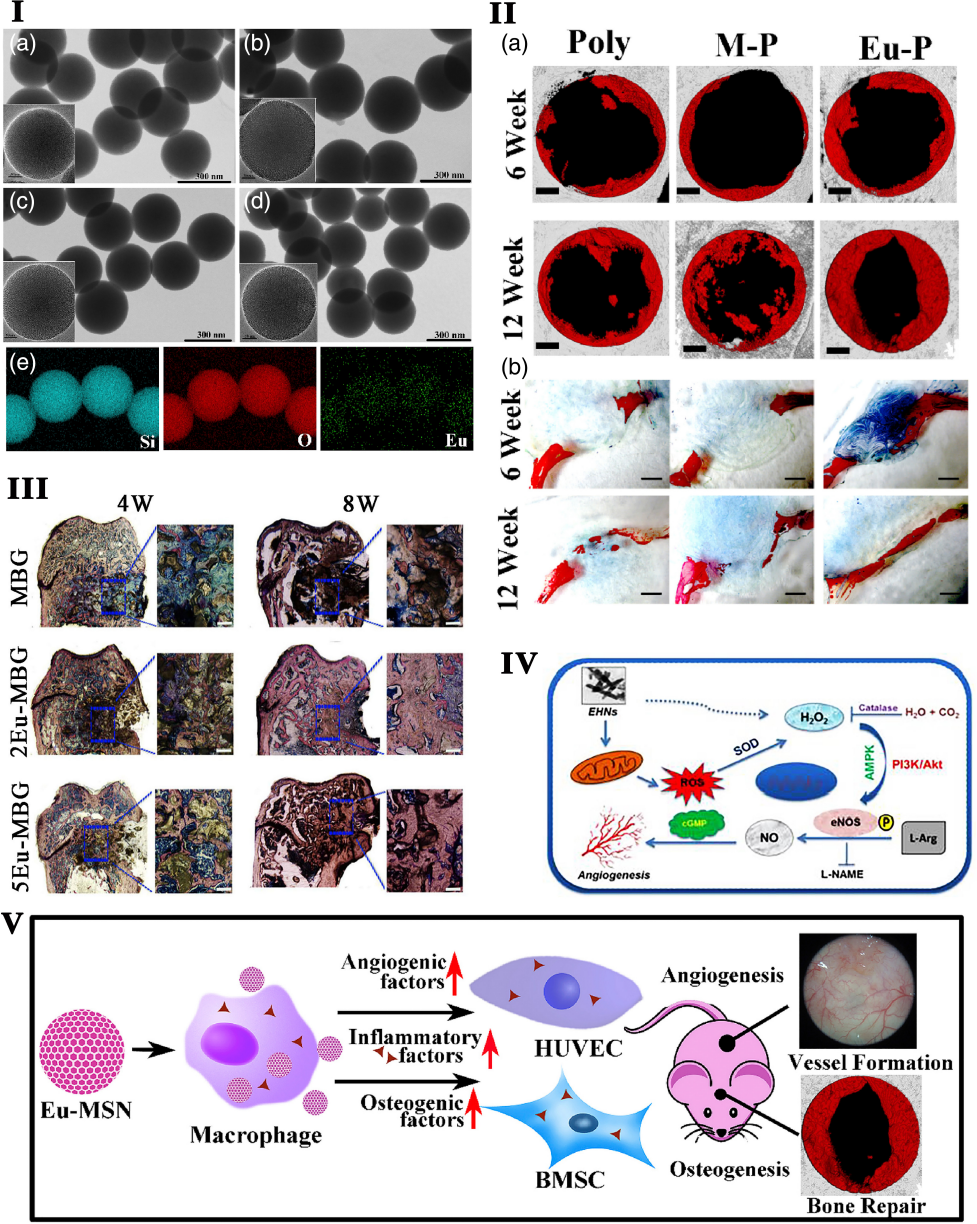
I. SEM images of pure MSNs (a), europium‐doped mesoporous silica nanospheres (1Eu‐MSNs) (b), 2Eu‐MSNs (c), and 3Eu‐MSNs (d) show uniformly spherical morphology with a size of 280–300 nm, and the inserted TEM images show the abundant mesoporous structure of nanoparticles. EDS mapping analysis (e) shows homogeneous element distribution of Si, O, and Eu in 2Eu‐MSNs typically. *Source*: Reprinted with permission from ref. 215. Copyright 2017, Elsevier. II. The effect of Eu‐MSNs on the in vivo osteogenesis. Representative micro‐CT images of new bone formation (the gray background represents normal skull, the black holes represent the cranial defect created by surgical operation with a diameter of 5 mm, and the red part represents the newly formed bone at the defect site, analyzed by CTAn software of micro‐CT) (a) in cranial defect at 6 weeks and 12‐week show larger new bone area in Eu‐MSNs‐polymer film (indicated as Eu‐P in figure) group. Immunofluorescent staining images (b) by VG stain in the cranial defects show that more new bone (red) was formed at the cross section of the defect in Eu‐P groups at 6 weeks and 12 weeks, indicating similar results as micro‐CT analysis (pure polymer film as Poly, MSNs‐polymer composite films as M‐P, and Eu‐MSNs‐polymer composite films with as Eu‐P), scale bar = 1 mm. *Source*: Reprinted with permission from ref. 215. Copyright 2017, Elsevier. II.) Osteogenic effect of europium. (a) Histological analysis and histomorphometric measurements of in vivo bone formation ability for MBG, 2Eu‐MBG, and 5Eu‐MBG scaffolds after implanted in the osteoporotic femur defects of OVX rats at 4 and 8 weeks. The scale bar is 100 μm. *Source*: Reprinted with permission from ref. 128. Copyright 2016, American Chemical Society. IV. Graphical representation of the hypothesized molecular mechanisms underlying the EHNs induced angiogenesis mediated through ROS‐NO‐cGMP signaling axis.^213^ *Source*: Republished with permission of Royal Society of Chemistry, 2015, permission conveyed through Copyright Clearance Center, Inc. V. The prepared Eu‐MSNs showed an inflammatory stimulation on macrophages, which further induced the osteogenic differentiation of bone marrow mesenchymal stem cells (BMSCs) via upregulating the gene expression of COL‐I, OCN, ALP, and RUNX2 as well as the angiogenic differentiation of HUVECs via upregulating the gene expression of CD31, MMP9, VEGFR, and PDGFR. The particles were then applied for in vivo experiments and showed a satisfactory effect on the bone repair of cranial defect and neovascularization. *Source*: Reprinted with permission from ref. 215. Copyright 2017, Elsevier. EHNs, europium hydroxide nanorods; HUVECs, human umblical vein endothelial cells; PDGFR, platelet‐derived growth factor receptor; SEM, scanning electron microscope; TEM, transmission electron microspcope

The gold nanoparticles conjugated with the europium luminescent probe and the peptide (pHLIP•EuL•Au) target the platelets in low pH 6.5 and translocate the pHLIP across the membrane.^212^ H_2_O_2,_ a redox signaling molecule generated by europium hydroxide nanoparticles, activates the endothelial nitric oxide synthase that promotes nitric oxide production in a PI3K (phosphoinositide 3‐ kinase)/Akt‐dependent manner, eventually triggering angiogenesis.^213^ The molecular mechanisms underlying the europium hydroxide nanorods (EHNs) induced angiogenesis are given in Figure 2IV. It has been further evidenced that microwave‐assisted synthesized europium (III) hydroxide nanorods exert pro‐angiogenic properties through ROS generation and activation of the MAPK pathway.^129^ On the other hand, Gd_2_O_3_:Eu^3+^ nanotubes generate excessive ROS injury to the mitochondria and DNA in BMSCs, and the release of cathepsin B by lysosomal rupture triggered cell death necrosis.^262^ The nanotubes of Gd_2_O_3_:Eu^3+^ remarkably enhance the bone mineral density and bone biomechanics as indicated by high ALP activity, mineralization and promoted the expression of osteogenesis genes in MC3T3‐E1 cells through activation of the BMP signaling pathway.^214^ Mesoporous bioactive glass (MBG) incorporated europium scaffolds by an in situ co‐template methods have highly interconnective large pores (300–500 μm), high specific surface area (140–290 m^2^/g), and well‐ordered mesopores (5 nm) as well as uniformly distributed europium elements. Incorporating 2–5 mol% europium toward MBG scaffolds with luminescent property stimulates new bone formation (Figure 2III) in osteoporotic bone defects in OVX rats.^128^

Figure 2I showed that the morphology of europium‐doped mesoporous silica nanospheres (Eu‐MSNs) stimulated the pro‐inflammatory response in macrophages, osteogenic differentiation of BMSCs, and angiogenic activity human umblical vein endothelial cells (HUVECs). Further, the Eu‐MSNs accelerate the new bone formation in the critical‐sized cranial defect site via immunomodulatory effect. The overall mechanism is provided in Figure 2II,V.^215^ Europium‐doped bioactive glass nanoparticles (BGNEu) significantly enhance human MSCs (hMSCs) osteogenic differentiation (ALP activity and COLI secretion) by activating osteogenic marker ALP, COLI, OPN, and RUNX2.^127^ Nanohydroxyapatite (nHAp) doped with Li^+^ ions (5 mol% Li+:nHAp) and co‐doped with lanthanide ions like samarium (III) (Sm^3+^) and europium (III) (Eu^3+^) ions enhance the luminescent property. Further, these composite improve osteogenic differentiation of human adipose‐tissue‐derived stem cells (hASCs) by a decrease in the expression of glycogen synthase kinase 3β (GSK3β) and an increase in β‐catenin mRNA level.^216^

### 5.3 Gadolinium

Gd occurs in many minerals and other RE materials, but it is obtained primarily from bastnasite. It was discovered by a Finnish chemist Johan Gadolin.^263^ Gd is known for its high potential in MRI. Nevertheless, its MRI applications are overshadowed by their large sizes resulting in poor organ/tumor targeting. Hsiao et al. used Gd as a dopant in fluorescein isothiocyanate mesoporous silica nanoparticles that possess green fluorescence and paramagnetism for labeling hMSCs via endocytosis. These labeled hMSCs can proliferate and differentiate into adipocytes, osteocytes, and chondrocytes.^167^ Further radiolabeled arginine‐glycine‐aspartic acid (RGD)‐functionalized Er^3+^/Yb^3+^ co‐doped NaGdF_4_ upconversion nanophosphors (UCNPs) had been developed to specifically target the α_v_β_3_ integrin‐expressing U87MG tumor cells and xenografted tumor models for tumor angiogenesis.^168^ Metallofullerenol Gd@C_82_(OH)_22_ effectively inhibits MMP‐2 activity by blocking the Zn21‐catalytic site directly or the S19 loop indirectly and inhibits the proteolysis of MMP‐9 via allosteric modulation with high antitumoral efficacy.^170^ The biocompatible dextran‐coated ultrafine sub‐10 nm Gd‐based nanoparticles are found particularly capable of determining the tumor boundary with clearly enhanced tumor angiogenesis.^169^

**FIGURE 3:**
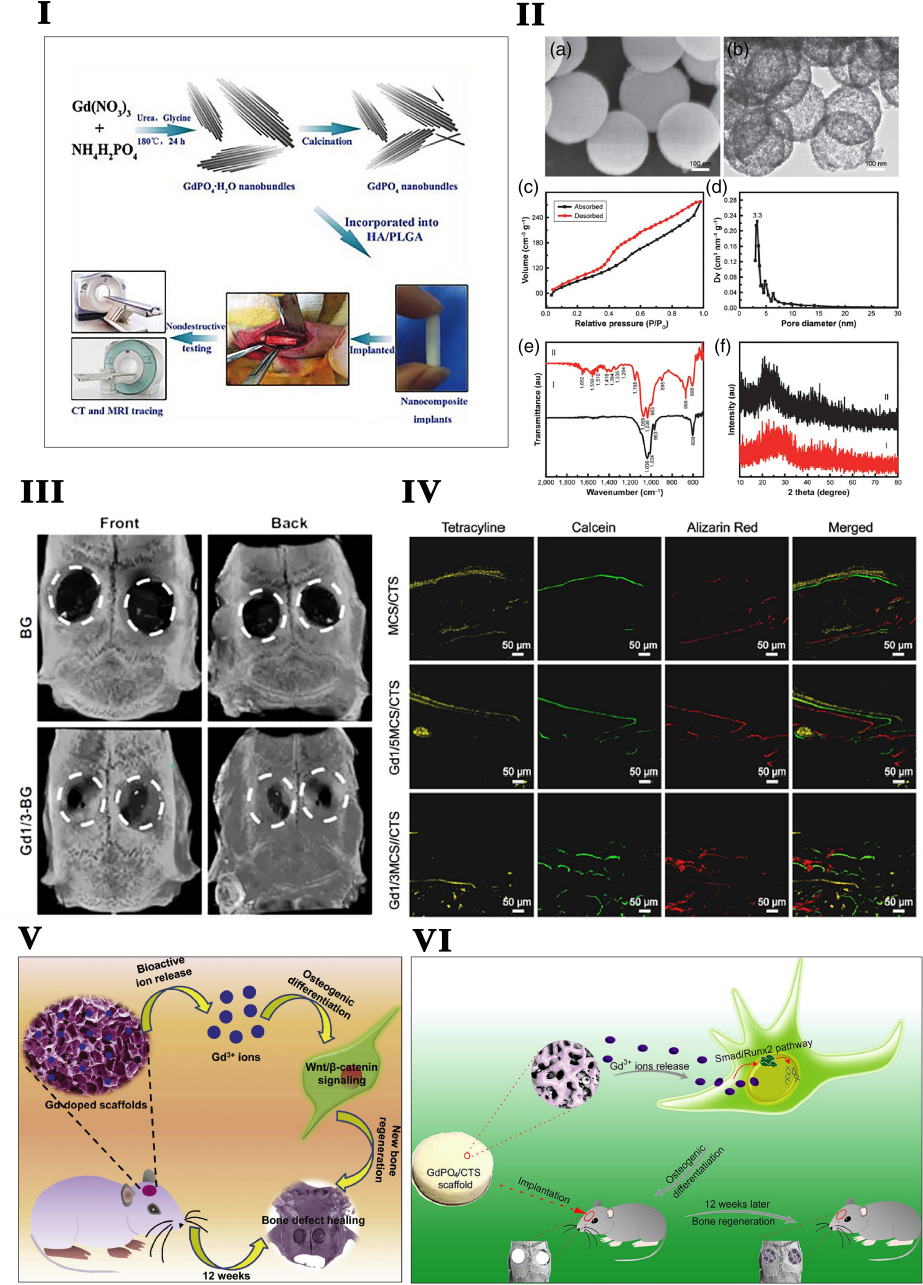
I. Schematic illustration of GdPO_4_·H_2_O and GdPO_4_ nanobundles synthesis and their application in biodegradable bone implants for MR and CT tracing. *Source*: Reprinted with permission from ref. 171. Copyright 2016, John Wiley & Sons, Inc. II. The structural property of the Gd‐BG scaffold. (a) SEM image and (b) TEM image of Gd‐BGS microspheres. (c) Nitrogen adsorption–desorption isotherm, (d) Barrett–Joyner–Halenda (BJH) pore‐size distribution curve of mesoporous Gd‐BGS microspheres. (e) The X‐ray diffraction patterns of samples: (I) Gd‐Bg microspheres and (II) Gd‐BG scaffolds. (f) The Fourier transform infrared spectra of samples: (I) Gd‐BG microspheres and (II) Gd‐BG scaffolds. *Source*: Reprinted with permission from ref. 217. Copyright 2019, Elsevier. III. Micro‐CT of rat cranial defects implanted with BG and Gd1/3‐BG scaffolds at 8 weeks after implantation. The images of reconstruction of micro‐CT for the bone regeneration of the defect area at Week 8. *Source*: Reprinted with permission from ref. 217. Copyright 2019, Elsevier. IV. Gd nanoparticle‐mediated bone tissue regeneration. Fluorochrome‐labeling analysis characterizing the new bone formation within MCS/CTS, Gd1/5MCS/CTS, and Gd1/3MCS/CTS scaffolds. Tetracycline (yellow), calcein (green), and alizarin red (red) were injected in rats at Weeks 3, 6, and 9. *Source*: Reprinted with permission from ref. 173. Copyright 2019, Elsevier. V. Schematic illustration of Gadolinium‐doped mesoporous calcium silicate/chitosan scaffolds enhanced bone regeneration ability. *Source*: Reprinted with permission from ref. 173. Copyright 2019, Elsevier. VI. Gadolinium phosphate/chitosan scaffolds promote new bone regeneration via Smad/Runx2 pathway. *Source*: Reprinted with permission from ref. 172. Copyright 2019, Elsevier. TEM, transmission electron microspcope

Solvothermal synthesized GdPO_4_H_2_O nanobundles incorporated HA and PLGA serve as a biodegradable and traceable bone implant for MRI and X‐ray tracing; this unique biomaterial promotes OCN expression in MC3T3‐E1 cells and bone mineralization in vivo rabbit radius defects (Figure 3I).^171^ GdPO_4_/chitosan scaffolds prepared by the lyophilization method improve the osteoconductivity, resulting in admired cell spreading and in vivo bone tissue in‐growth. GdPO_4_ nanoparticles in the GdPO_4_/CTS scaffolds robustly promote osteogenic differentiation by upregulating the levels of ALP, RUNX2, OCN, and COLI expression in rabbit BMSCs via activation of the Smad/RUNX2 signaling pathway (Figure 3VI).^172^ Gd‐doped MCS/CTS (Gd‐MCS/CTS) scaffolds show anabolic effects on rabbit BMSCs cell proliferation and osteogenic differentiation through the activation of the Wnt/β‐catenin signaling pathway (Figure 3IV,V).^173^ Gd‐BG scaffolds promote the proliferation and osteogenic differentiation of human BMSCs via the Akt/GSK3β signaling pathway (Figure 3II,III).^217^ Gd is a widely accepted contrast agent in MRI, cardiac applications such as effective MR angiography.^264^ Gd ethoxybenzyl diethylenetriamine pentaacetic acid (Gd‐EOB‐DTPA) is the liver‐specific contrast enhancement agent presently used for diagnosing HCC. MRI with Gd‐EOB‐DTPA enhancement is superior to enhanced CT and conventional contrast‐enhanced MRI in diagnosing small liver lesions and differentiating benign and malignant nodules. Gd‐EOB‐DTPA excretes into the biliary tract through multidrug resistance‐associated protein 2 (MRP2) on the biliary tract. The period of this phase is called the hepatobiliary specific period or hepatobiliary phase. The remaining contrast agent, similar to Gd‐DTPA, can be excreted through the kidney. This dual clearance pathway can compensate for each other when the liver or kidney function is damaged, thereby ensuring higher safety.^265^ Compared with conventional hepatobiliary MRI, enhanced MRI by Gd‐BOPTA combined with ultrasound has good diagnostic value in determining HCC.^266^ Gd(III) complexes containing a polydentate carboxylate ligand exhibit good MRI contrast properties.^267^ PEGGd_2_O_3_ NPs presented longer half‐life, similar acute toxicity and histological influence, more negligible effect on hepatic and renal functions, and stronger contrast enhancement in the tumor.^268^ Gd_2_O_3_‐assembled mesoporous silica MCM‐41 nanocomposite has been identified both in vitro and in vivo as a safe MRI contrast medium with better efficacy than its commercially available counterpart Gd‐DTPA.^269^ An ultrasmall, theranostic (3.0 ± 1.0 nm size) Gd‐based nanoparticle (AGuIX NPs) are used to improve radiographic delineation and increase the intratumoral dose‐effect delivered by the particles.^270^

Further, it has been used as an MRI or X‐ray contrast agent of the osteoblasts applied in biodegradable HA/PLGA bone implants in vivo, providing a practical approach for recognizing the implants or the newly formed bone tissues.^171^ GD MRI enhancer‐based dynamic contrast‐enhanced (DCE) MR examinations at 3 T assess perfusion in healthy carpal bones in a patient with osteonecrosis and Kienbock's disease.^218^ The results suggested that areas of healthy bone show low perfusion. DCE‐MRI at 3 T diagnoses altered perfusion in patients with Kienbock's disease. RE element Gd‐doped magnesium scaffold (CaP‐coated Mg‐Zn‐Gd) enhances orthotopic reconstruction of large‐sized orbital bone defect healing in canines. The scaffolds triggered trigeminal neurons via CGRP promote endomucin expression in endothelial cells, facilitating angiogenesis and osteogenesis.^101^ Gd (III) oxide nanoparticles (70 nm size) synthesized via the polyol method and surface functionalized with a bisphosphonate (BP) derivative (GBCAs)‐BP) show a strong affinity towards calcium phosphate. The CPC‐GBCAs‐BP functional material is longitudinally monitored after in vivo implantation in a condyle defect rat model. The BP functionalization prolongs the residence of the contrast agent within the CPC to allow long‐term follow‐up imaging studies.^131^ Heat shock protein 16.5 (Hsp16.5) and peptide conjugated Gd (III) nanocages detect neuropilin‐1‐positive cells in genetically engineered mouse models.^219^ Papageorgiou et al. used Gd for whole‐body magnetic resonance imaging, a radiation‐free alternative to the 99mTc‐HDP bone scan (BS) to detect metastasis of cancer bone.^130^ Since Gd‐based contrast agents (GBCAs) are used for MRI enhancers in the bone; it has some adverse effects on the body. For instance, Gd concentration in bone is significantly higher in exposed subjects than in control subjects. Gd can be retained in bone up to 5 years after one GBCA administration.^271^ The Gd‐exposed tibia shows a higher Gd concentration compared to the control group.^272^ Based on the reports mentioned above from the literature, Gd can be used not only for the bone regeneration application but also to visualize the damaged bone and newly formed bone in vivo.

### 5.4 Neodymium

Neodymium is a ductile and malleable silvery‐white metal. Austrian chemist Carl Auer von Welsbach discovered neodymium in 1885. Neodymium occurs in the least amount in the rocks of Earth's crust. The major application of neodymium is in high‐strength permanent magnets used in high‐performance electric motors and generators, the electronics industry, and the ceramics industry for glazes and color glass in various shades from pink to purple. Neodymium‐stabilized yttrium aluminum garnet (YAG) is a component of many modern lasers, and neodymium glasses are used in fiber optics.^273^ Neodymium is used in a laser oscillator to irradiate the specimen. Nd:YVO_4_ laser oscillator has a threshold average laser power of 160 mW required to drill through a 0.75‐mm thick cortical bone with a peak intensity of 1.3 GW/cm^2^.^223^ Nd‐YAG laser irradiation in the near‐infrared ray (NIR) area has been reported to promote bone healing via the expression of ALP, RANKL, and OPG. It indicated that osteoblast‐like cells activate genes related to bone metabolism by combining mechanical stimulation and laser irradiation.^274^ Nd:YAG laser irradiation stimulates cell growth in the nonsensitized osteoblasts and induces the expression of osteopontin, ALP, and RUNX2 in osteoblasts, type I COLI in fibroblasts, and vinculin in endothelial cells in low pulse energy levels.^275^ Nd:YAG laser treatment improves zirconia bioactivity by increasing human osteoblast's cell viability, proliferation, and expression of COL1 and ALP activity.^276^ Nd:YAG is frequently used as an alternate nonsurgical mechanical debridement of peri‐implant diseases. Single time Nd:YAG laser treatment effectively decreases the peri‐implant inflammatory parameters plaque index, bleeding on probing, and probing depth indicated that Nd:YAG laser‐assisted nonsurgical MD is more effective in reducing peri‐implant soft tissue inflammatory parameters than MD alone in the short term but not in long term.^277^ The major challenge for orthodontic treatments lies in moving the tooth and shortening the time. Nd:YAG laser irradiation on orthodontic tooth movement with 1064 nm stimulates osteoblasts via producing ROS and nitric oxide. A higher RANKL/OPG ratio leads to the activation of osteoclasts. Higher RANKL expression was observed in the prolonged laser irradiation side, while no change was noticed in the expression of OPG.^228^ It has been found that the Nd:YAG laser irradiation of bone for the long term severely delays bone healing as compared to positive control bur osteotomy sites and in patients with osteopenia or osteoporosis.^133^, ^278^ So the slight modification of Nd:YAG laser with silicon carbide on titanium‐6 aluminum‐4 vanadium (Ti6Al4V) alloys had been prepared to promote the osteoblast cell growth effectively.^224^ To exterminate the delayed bone healing induced by Nd:YAG, Kim et al. use high‐power, low‐level Nd:YAG laser, which increases osteoblast activity very efficiently, accelerating the mineral deposition via activation of the BMP‐2‐related signaling pathway in MC3T3‐E1 osteoblasts.^132^ A pulsed Nd:YAG laser is an effective physiotherapy modality used as a Class IV high‐intensity laser therapy combined with exercise, which effectively increases lumbar and total hip BMD after 24 weeks of treatment, with effects lasting up to 1 year. High‐intensity, pulsed, and high‐power laser irradiation applied once every 2 days for 2 weeks effectively enhanced bone regeneration in an osseous defect in rats. The power magnitude did not affect the osseous regeneration process but was presumed to be more efficient at the dose of 0.75 W, lower than 3 W. These data indicated that the Nd:YAG laser light could heal local bone loss after surgical treatment.^278^

**FIGURE 4:**
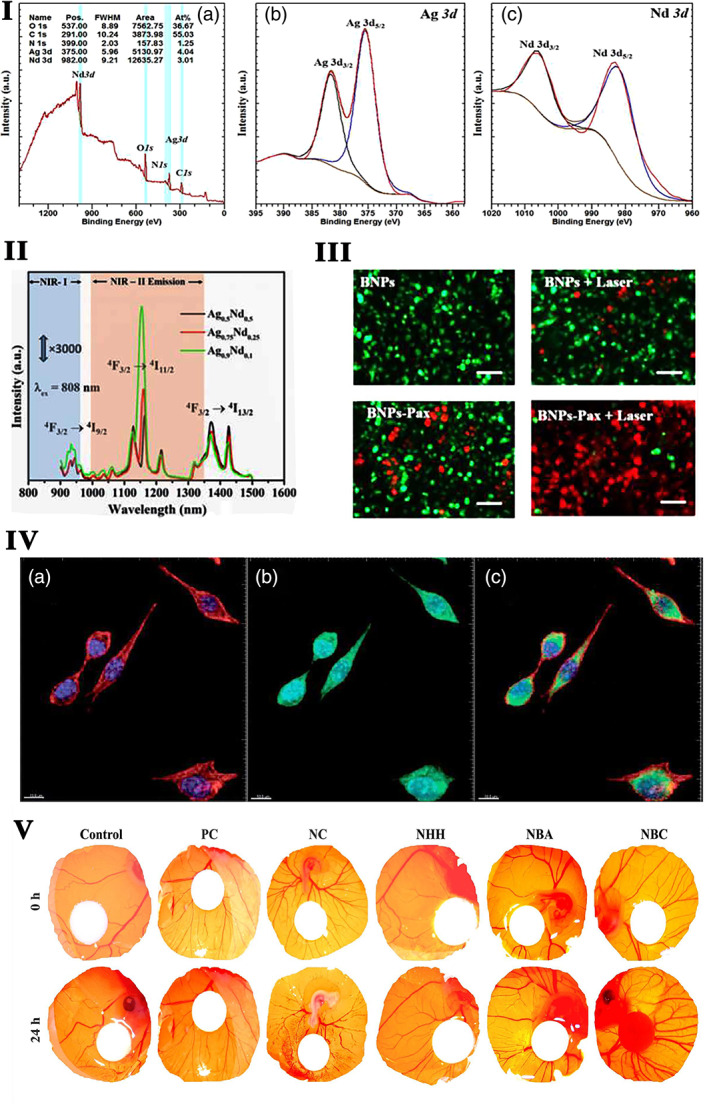
I. Photoemission spectra of BNPs: (a) survey spectra and high‐resolution spectra of (b) Ag 3d (c) Nd 3d. II. Emission spectra of Ag−Nd BNPs on excitation with 808 nm reveals mission ability in the NIR (750–1600 nm) region, with strong emission in the region of the second biological window, which is more transparent for deep tissue penetration. III. Fluorescence images of treated cells (scale bar = 100 μm). *Source*: Reprinted with permission from ref. 279. Copyright 2017, Elsevier. IV. Bimodal imaging by rare‐earth nanoparticles. Multiphoton microscopy images of fibroblast cells with PMAO coated GdF_3_:Nd^3+^ nanoparticles. (a) Image of the DAPI‐stained nuclei (blue channel) and phalloidin‐stained cytoplasm (red channel). (b) Observed emission of the nanoparticles under 488 nm excitation. The green color denotes emission correlated with the cytoplasm, and the light blue color denotes emission correlated with the nuclei. (c) Images of the DAPI, phalloidin, and fluorescent channels together. *Source*: Reprinted with permission from ref. 280. Republished with permission of Royal Society of Chemistry, 2013, permission conveyed through Copyright Clearance Center, Inc. V. The angiogenic property of Nd nanopolymorphs assessed using the chorioallantoic membrane (CAM) chick egg model. PC, positive control (20 ng VEGF‐treated CAM), NC, negative control (200 μM thalidomide‐treated CAM), NHH, Nd nanoparticles, NBA, Nd nanocubes, NBC, Nd nanorods. *Source*: Reprinted with permission from ref. 18. Copyright 2019, Elsevier

Enamel matrix proteins (EMPs) are widely used in periodontal surgery for the regeneration of periodontal tissues. The use of Nd:YAG laser with EMP heals the intrabony defects of periodontal disease. This treatment approach decreases the probing depth and increases the clinical attachment level compared to baseline values.^225^ Similarly, Nd:YAG laser in combination with scaling and root planning (SRP) alleviates periodontal inflammatory parameters plaque index, gingival index, and probing pocket depth, as well as reduces marginal bone loss compared to treatment by SRP alone.^226^ The nanophosphors of GdF_3_:Nd^3+^ coated with poly(maleic anhydride‐alt‐1‐octadicene) (PMAO) have no significant cellular toxicity for concentrations up to 200 mg ml^−1^. Furthermore, the incorporation of Gd into the nanocrystalline structure makes an ideal structure for use as MRI contrast agents (Figure 4IV).^280^ Rocha et al. found that neodymium‐doped LaF_3_ core/shell nanoparticles emerge as relevant sub‐tissue optical probes for bioimaging.^281^ Further experiments from their team reported that Nd^3+^‐doped LaF_3_ (Nd^3+^:LaF_3_) nanoparticles exhibit fluorescence in three main emission channels of Nd^3+^ ions like 910, 1050, and 1330 nm, respectively. The optimal fluorescence of Nd^3+^‐doped LaF_3_ nanoparticles in terms of relative emission intensities, penetration depths, and sub tissue optical dispersion is higher in 4F_3/2_→4I_11/2_ (1050 nm in the second biological window) than the 4F_3/2_→4I_9/2_ (910 nm, in the first biological window).^282^

Nano‐sized neodymium oxide (Nd_2_O_3_) arrests the S‐phase of the cell cycle, disrupts mitochondrial membrane potential, and inhibits proteasome activity, leading to autophagy in non‐small cell lung cancer NCI‐H460 cell.^283^ Microwave‐assisted polyol‐based chitosan‐functionalized silver‐neodymium bimetallic nanoparticles (Ag‐Nd BNPs, 10 nm) exhibit fluorescence in the NIR region and magnetic properties (Figure 4I,II). Ag‐Nd BNPs had excellent biocompatibility and also promoted the loading of the anticancer drug paclitaxel. The synergistic effect of paclitaxel and the photothermal property enables Ag‐Nd BNPs to destroy cancer cells in vitro at a low dose compared to single therapy (Figure 4III).^279^ Nd‐diethylene triamine penta acetate acid (Nd‐DTPA) complex shows bright narrow‐band emission at 1330 nm for in vivo NIR‐II bioimaging with rapid renal excretion and high biocompatibility and optical‐guided small tumor (down to ~3 mm) detection.^284^ Polyacrylic acid (PAA)‐modified NaLuF_4_:Gd/Nd nanorods are used in tiny tumor detection. The NIR‐II emission at 1056 nm and 1328 nm with high photostability of Nd can utilize for NIR‐II optical imaging of small tumor (5 mm) diagnosis and small blood vessel with a high resolution (~105 μm).^285^ Recently, Ma et al. prepared implantable multifunctional material of Nd‐Ca‐Si silicate glasses and glass/alginate composite hydrogels, which have photothermal properties with unique temperature monitoring, photothermal function, and wound healing bioactivity that can be used for localized thermal therapy for cancer treatment. Besides, the composite hydrogel has bioactivity to repair heat damage‐caused wounds by PTT due to the bioactive silicate components.^230^ These findings demonstrate that the explored lanthanide‐based probes are promising NIR contrast agents for future biomedical applications, such as early diagnosis of a small tumor, vascular‐related disease imaging, angiogenesis, and diagnosis. Recently Ansari et al. reported that surface‐modified mesoporous silica micro‐cocoon with neodymium hydroxide (Nd(OH)_3_) shows good cell viability even at high concentrations and hydrophilic conditions. These nontoxic cocoon‐shaped microstructures could be potentially suitable candidates for optical bio‐probes and drug delivery applications.^286^ Nd_2_O_3_ exposure on human bronchial epithelial cells (16HBE) initiates an inflammatory response via the p‐STAT3 pathway.^227^ Nd_2_O_3_‐treated 16HBE cells release the pro‐inflammatory cytokines IL‐6 and IL‐8 and upregulate circRNA 0039411 (circ_0039411) by sponging miR‐93‐5p.^227^ These anticancer applications of RE smart nano‐biomaterials might be helpful to combine with the osteogenic treatment during cancer metastasis‐induced bone loss.

Our previous research revealed that neodymium nanoparticles exhibit a redox‐mediated angiogenic response in a shape‐dependent manner (Figure 4V). The redox signaling perceived via PKM2‐NOX4 signaling pathways activates the pro‐angiogenic factors, namely, VE‐cadherin, HIF1α, VEGF, and VEGFR2, to facilitate the angiogenic process in EA. Hy 926 cells.^18^ The static magnetic field of neodymium is helpful to promote the bone formation faster after the bone is wounded. The implant stability quotient values and tissue response after implant placement under the influence of the magnetic field are significantly higher than on the nonmagnetic side. A positive correlation has existed between the magnetic field and osseointegration.^287^ Nd:YAG laser irradiation significantly enhances the amount of orthodontic tooth movement, the expressions of ALP and RANKL at the pressure site, and no difference in OPG expression.^228^ These effects stimulate osteoclast and osteoblast activation and accelerate bone metabolism during tooth movement. The laser melting method alloyed neodymium with Mg‐5.6, Zn‐0.5, and zirconia enhances corrosion resistance and exhibits excellent biocompatibility.^288^ Shreds of evidence revealed that microorganisms play the chief role in causing peri‐implantitis. Short pulse laser‐induced by Nd:YAG Q‐switch laser in nanoseconds cleans contaminated implant surfaces to treat peri‐implantitis significantly.^229^

Besides the application in laser irradiation, bone healing, and bioimaging, the nanoparticles of neodymium (III) hexacyanoferrate (II) (NdHCF) coated on the surface of carbon paste electrode are used for sensing the glucose by enzymatic reaction of the glucose oxidase (GOx) with NdHCF.^289^ Pourjavid et al. developed the highly selective Nd(III) PVC‐based membrane sensor with sodium tetraphenylborate (NaTPB) and oleic acid (OA) as anionic additives and benzyl acetate (BA), dibutyl phthalate (DBP), o‐nitrophenyloctyl ether (NPOE), and acetophenone (AP) as plasticizing solvent mediators to trace Nd (III) ions in some binary mixtures such as mouth washing solutions, soil, and sediment samples.^290^ Further, neodymium and fluorine‐doped TiO_2_ act as a photocatalyst, which increases the rate of methylene blue degradation to about 1.76 and 1.45 times higher than undoped TiO_2_ in ultraviolet light and visible light, respectively.^291^

### 5.5 Lanthanum and other RE metal‐doped nano‐biomaterials

**FIGURE 5:**
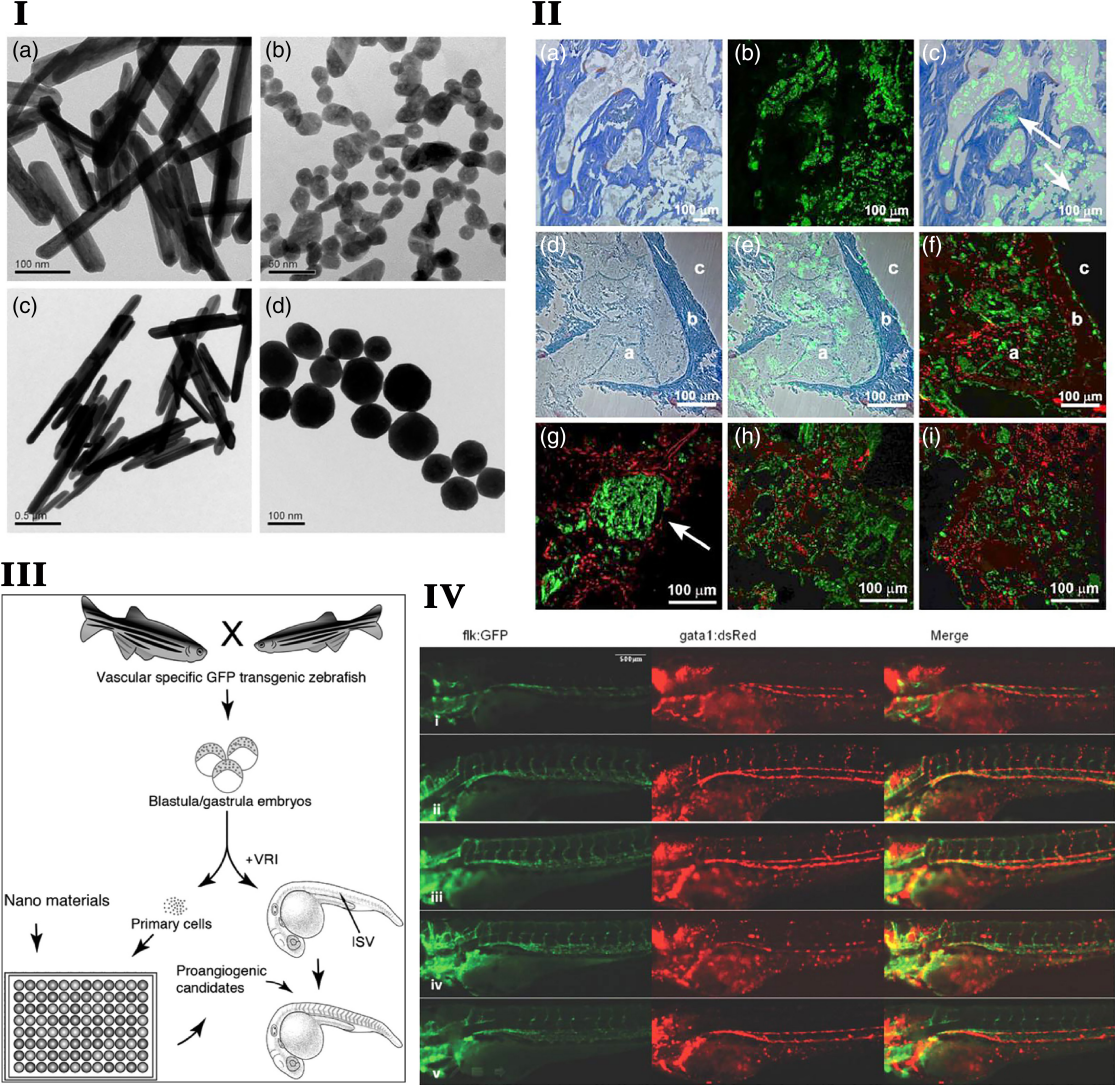
I. Representative TEM images of nanoparticles with proangiogenesis activity. (a) Eu rods, (b) Eu spheres, (c) Tb rods, and (d) Tb spheres. *Source*: Republished with permission of Royal Society of Chemistry. Reprinted with permission from ref. 295. Copyright 2016, Copyright Clearance Center, Inc. II. Yb^(3+)^/Ho^(3+)^ co‐doped apatite upconversion nanoparticles to distinguish implanted material from bone tissue. (a) The light image of the Masson's stained histological section of new bone tissue (matured: blue, growing: red). (b) The upconversion green fluorescent image of the implanted FA:10Yb^3+^/0.5Ho^3+^ particles. (c) Their overlap image after 4 months. (d) The light image of the stained new bone tissue after 6 months. (e) Overlapping image of the light image and the upconversion green fluorescent image of the implanted FA:10Yb^3+^/0.5Ho^3+^ particles. (f) The superposition of the red fluorescent image of the new bone tissue under 561 nm laser excitation and the green fluorescent image of the FA:10Yb^3+^/0.5Ho^3+^ particles under 980 nm NIR excitation. The confocal superposition images of FA:10Yb^3+^/0.5Ho^3+^ particles (green) and new bone tissue (red) at 2 (g), 4 (h), and 6 (i) months after implantation. *Source*: Reprinted with permission from ref. 220. Copyright 2016, American Chemical Society. III. Schematic diagram showing the overall strategy and methodology of our experiments illustrating Tg(flk: EGFP) transgenic primary cell and whole embryo‐based high‐throughput screening for nanomaterials with proangiogenesis activity. *Source*: Reprinted with permission from ref. 295. Copyright 2016, Royal Society of Chemistry. IV. Lanthanide nanoparticles could recover circulation in VRI pretreated zebrafish embryos. Zebrafish embryos at 72 hpf. (i) Blank control, (ii) 100 μg ml^−1^ Eu rods, (iii) 100 μg ml^−1^ Eu spheres, (iv) 100 μg ml^−1^ Tb rods, and (v) 100 μg ml^−1^ Tb spheres. The green channel represents the blood vessels, while the red channel represents the mature blood cells. The merged pictures indicate that the embryonic circulation in the ISV region has recovered after the treatment of nanoparticles in this method. *Source*: Reprinted with permission from ref. 295. Republished with permission of Royal Society of Chemistry, 2016, Copyright Clearance Center, Inc. SEM, scanning electron microscope; TEM, transmission electron microspcope

Lanthanum is the second most reactive and malleable silvery‐white rare‐earth metal. Lanthanum was discovered in 1839 by Carl Gustaf Mosander. Lanthanum occurs in the rare‐earth minerals monazite and bastnasite. Lanthanum compounds are used as hosts for phosphors in fluorescent lighting and X‐ray detectors.^292^ Lanthanum oxide nanoparticles (LONPs) exert their action via the release of ROS. LONP extracts do not exert any acute systemic toxicity effects in mice. On the other hand, LONP exerts toxicity to the liver following oral administration, suggesting that these particles are absorbed from the gastrointestinal tract and deposited in the hepatobiliary system. LONP did not show any mutation in the Ames test, both in the presence or absence of S‐9.^293^ The accumulation of lanthanides in hepatocytes gradually increases dose dependent with exposure to the elements like La and Ce. These lanthanides enter hepatocytes and accumulated in the nuclei, and induce oxidative damage in hepatic nuclei and mitochondria, as indicated by decreased levels of SOD, CAT, and GSH.^294^ Hydrothermally prepared Yb^3+^, Ho^3+^ co‐doped fluorapatite (FA:Yb^3+^/Ho^3+^), and hydroxyapatite (HA:Yb^3+^/Ho^3+^) particles exhibited green (FA:Yb^3+^/Ho^3+^) and red (HA:Yb^3+^/Ho^3+^) upconversion emissions under 980 nm near‐infrared excitation due to its lattice structure and composition. The upconversion apatite particles are used to distinguish implanted material from bone tissue. An image superposition method provides a novel strategy for long‐term fluorescence tracking of implanted material or scaffold during bone regeneration (Figure 5II).^220^ Magnetic lanthanum‐doped HA/CS scaffolds recruit rat BMSCs and modulate host‐to‐scaffold immune responses by promoting M2 macrophage polarization in vitro by upregulating the phosphorylation of the Smad 1/5/9 pathway that eventually promote bone regeneration.^92^ Furthermore, lanthanum‐doped scaffolds promote osteogenic differentiation of bone marrow mesenchymal stem cells (BMSCs) through the Wnt/β‐catenin signaling pathway and induce high expression of the osteogenic markers and enhance bone regeneration in rat critical‐sized calvarial defect sites.^221^ Another study reported that La^3+^ ions in the bone scaffolds remarkably induce the osteogenic differentiation of rabbit BMSCs via the activation of the TGFβ signaling pathway.^222^

Among 17 RE elements, the osteogenic and bone defect healing potential of only a few RE elements had been extensively explored. Bone regeneration related‐biological functions of other RE metal‐based nanomaterials are reported sporadically. Radiolabeled arginine‐glycine‐aspartic acid (RGD)‐functionalized Er^3+^/Yb^3+^ co‐doped NaGdF_4_ upconversion nanophosphors (UCNPs) had been developed to specifically target the α_v_β_3_ integrin‐expressing U87MG tumor cells and xenografted tumor models for tumor angiogenesis.^168^ It has been reported that Eu III(OH)_3_ and TbIII(OH)_3_ promote angiogenesis in the transgenic zebrafish model. (Figure 5I,III,IV)^295^ Zou et al. reported that the one‐pot hydrothermal carbonization method synthesized praseodymium co‐doped carbon quantum dots (Ce/Pr‐C GR‐HA) enhance hydroxyl radical scavenging property with favorable biocompatibility and negligible cytotoxicity. These carbon dots are readily internalized into the cytoplasm and decrease ROS level.^186^

Further radiolabeled arginine‐glycine‐aspartic acid (RGD)‐functionalized Er^3+^/Yb^3+^ co‐doped NaGdF_4_ UCNPs had been developed to specifically target the α_v_β_3_ integrin‐expressing U87MG tumor cells and xenografted tumor models for tumor angiogenesis.^168^ Samarium‐doped YVO_4_ nanoparticles (20–50 nm) show significant toxicity in RAW 264.7 macrophages at concentrations of 25 mg/ml than erbium‐doped YVO_4_.^188^ Ethylenediamine tetramethylene phosphonic acid (EDTMP), and technetium‐99m‐labeled samarium nanoparticles accumulate in the bone tissue for extended periods (150 min), resulting in the prolonged release of EDTMP at the target site. This prolonged release may be a more optimal treatment for the management of cancer bone metastasis‐related pain.^189^ Morais et al. fabricated samarium (Sm^3+^)‐doped P_2_O_5_ glass‐reinforced HA‐based bone composites, which enhance the F‐actin cytoskeleton organization and cell proliferation and expression of relevant osteoblastic genes. Also, Sm^3+^ doping reduces the adhesion of *S. aureus* and *S. epidermidis* on bone substitutes. The improved osteoblastic behavior and the antibacterial effects are dependent on the amount of samarium in the composite.^231^ Augustine et al. reported that Y_2_O_3_ nanoparticles incorporated polycaprolactone scaffolds promote the expression of cell proliferation and angiogenesis‐related markers such as VEGF and endothelial growth factor receptor (EGFR) in fibroblasts (L‐929) and osteoblast‐like cells UMR‐106.^232^

Erbium:YAG (Er:YAG) laser‐assisted bone irradiation promotes inflammatory cell infiltration, fibroblastic reaction, and revascularization adjacent to the irradiated bone surface.^296^ Even though Er:YAG is being used in clinical practice, the water content of bone usually changes with the position. At the same time, the amount of water spray in the process of laser irradiation is also uncertain. In order to avoid this problem, Huang et al. used optical coherence tomography (OCT) to characterize the roughness and thickness of the heterogeneous layer on the cortical bone surface with different moisture contents that led to different ablation effects. The results from their study showed that OCT could quickly and accurately evaluate the differences between the moisture content, as compared to histology and scanning electron microscope (SEM).^233^ NaYF_4_:Yb, Er@CaF_2_ nanoparticles with a small size (10–13 nm) robustly enhance (ca. 300 times) upconversion emission compared with the pristine nanoparticles. The CaF_2_ shell protects the rare‐earth ions from leaking when the nanoparticles are exposed to the buffer solution and ensure biological safety for the potential bio probe applications.^13^ Nanoparticle‐based in vivo imaging is hindered by the autofluorescence of the host cells and tissues. This issue could be addressed by the use of HA:Yb/Ho as an upconversion material. Ytterbium (Yb) and holmium (Ho) co‐doped HA matrix favors by its bright fluorescence under NIR irradiation and enhances bone formation.^297^ Another study conducted by Nethi et al. extensively studied the pro‐angiogenic properties of terbium hydroxide nanorods. They reported that the pro‐angiogenic property of Tb enhances wound healing in mouse models.^191^

## 6 CHALLENGES OF RE ELEMENTS AND THEIR USE IN BONE REGENERATION

**FIGURE 6:**
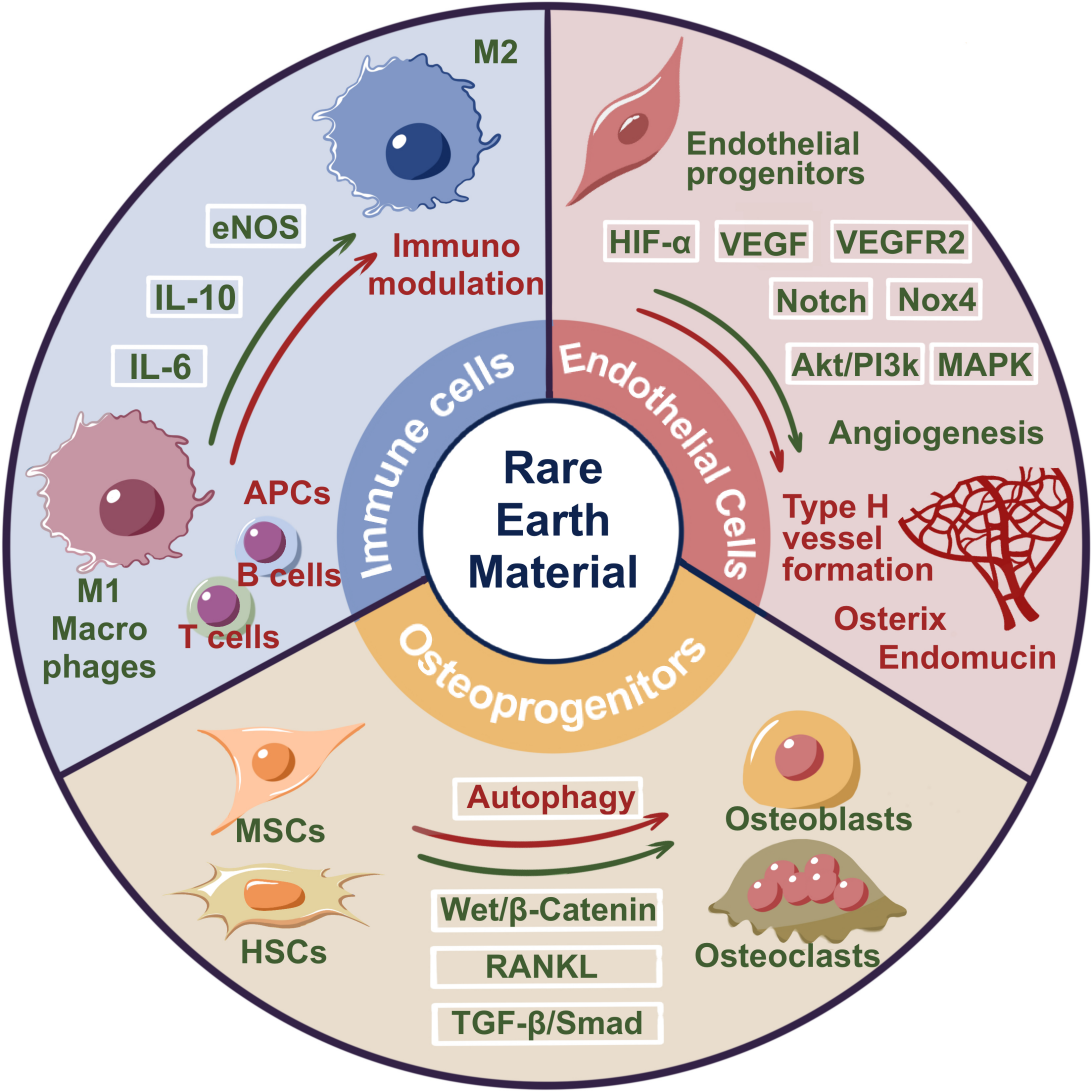
Advances and prospects of molecular mechanisms involved in RE smart nano‐biomaterial‐based bone tissue engineering and implant osseointegration. Green color text and arrows indicate the already explored mechanisms, and the red color text and arrows indicate the possible mechanisms that need to be explored

**FIGURE 7:**
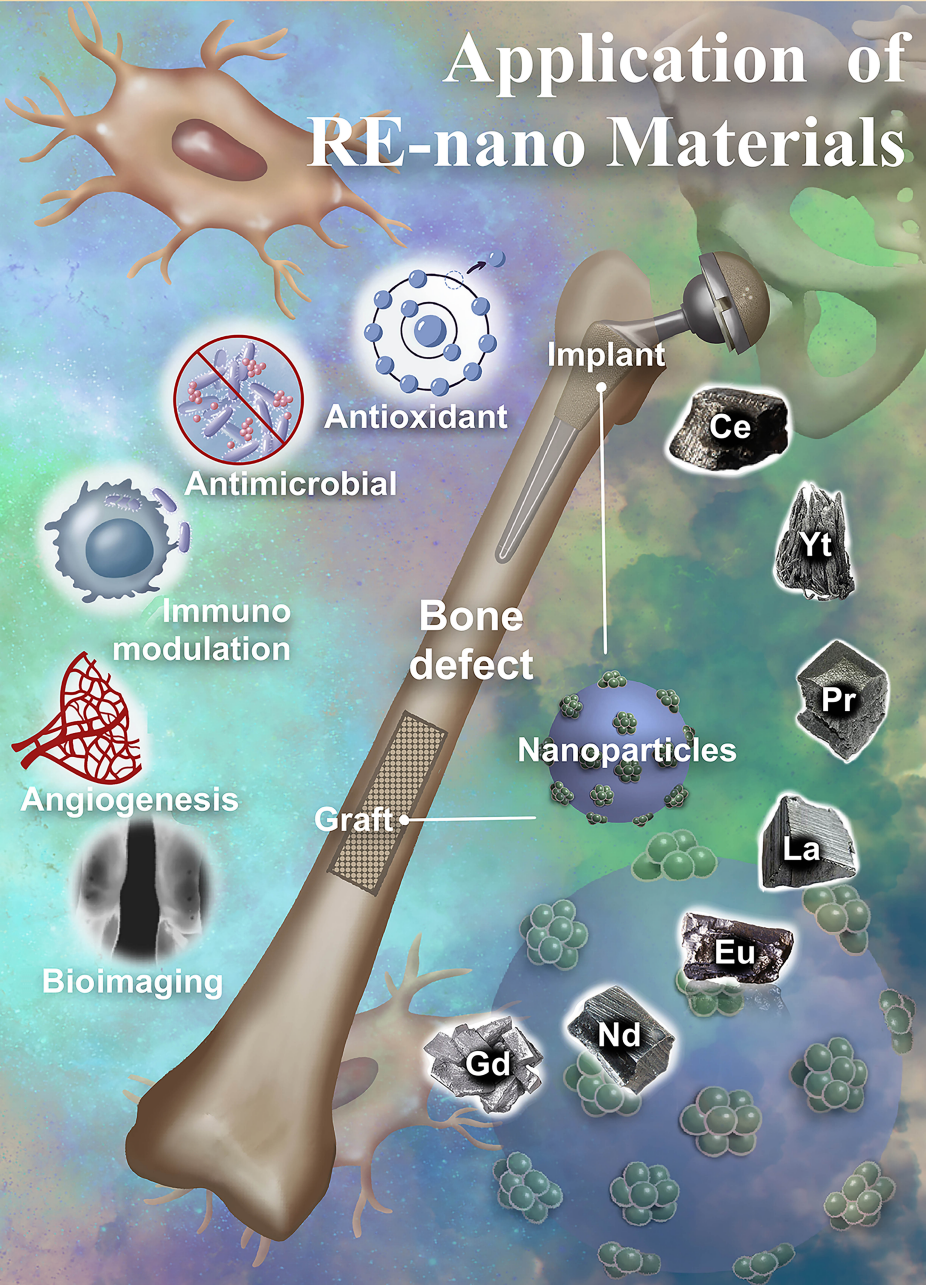
Potential applications of RE biomaterials in bone tissue engineering and implantology

RE elements hold unique biological properties required for effective bone regeneration, such as pro‐angiogenic, immunomodulatory, antimicrobial, and osteogenic.^18^, ^129^, ^197^, ^280^ The various biological processes and signaling molecules involved in RE material‐mediated bone defects healing are depicted in Figure 6. The advances in RE nano‐biomaterials for bone tissue engineering and implantology are aforementioned in this review. Overall, potential applications of RE materials in bone tissue engineering and implantology are depicted in Figure 7. RE materials in bone tissue engineering and bone defect healing in the clinic are still a long way to go. One of the major challenges in RE material‐based bone regeneration is progenitor cells' recruitment and biological activity. Some RE nanomaterials are engaged in recruiting immature progenitor cells like MSCs and stimulating them to develop osteoblasts, mediated by a cascade of signals and the activations of several extra and intracellular receptors. The recruitment of progenitor cells is mainly regulated via epigenetic, cellular reprogramming, cell metabolism, and autophagy.^298^ It has been reported that decreased level of autophagy in human MSCs reduces osteoblast differentiation.^299^ The molecular mechanisms involved in RE material‐induced autophagy in bone cells are not yet fully elucidated. The recruitment and activation of immune cells are essential for effective and accelerated bone fracture healing. RE nanomaterials had been reported to modulate macrophage polarization during bone defect healing. However, the effect of RE nanomaterials on the expansion and activation of various immune cells regulating bone homeostasis, including T cells, B cells, and neutrophils, has not been investigated yet.

Furthermore, in the bone fracture microenvironment, the ROS levels are abundantly high and affect bone reconstruction.^300^ Excessive ROS production can induce osteoclastogenesis,^57^ whereas hydrogen peroxide suppresses the osteoblastic differentiation process in primary mouse BMSCs.^301^ There is an opposing role of RE materials in producing reactive oxygen species and altering the redox states in the bone defect site. Thereby, it is inevitable to tune or modulate the redox signaling intersecting the current problem. Most of these studies lack the in‐depth investigation on local and systemic adverse effects of in vivo applied RE nano‐biomaterials in long‐term use. Therefore, designing suitable graft materials and optimizing the proper dose of RE material to stimulate biological functions required for bone regeneration is the most challenging. The clinical usage of rare‐earth‐based materials in the tissue engineering field is restricted by a lack of site specificity and sustained delivery of RE elements. Direct injection of nanomaterials in the minor defects and fracture sites and systemic injection targeting osteoporotic bone/defect sites are under investigation.^302^, ^303^, ^304^ Whereas nonunion fractures and critical‐sized bone defects need specialized treatment modalities. Mainstream reports from the literature had indicated the in vitro and in vivo osteogenic properties of RE nanoparticles. However, literature has indicated the inhibitory effect of RE nanoparticles on cell viability and osteogenic potential.^77^, ^203^, ^257^ This inhibitory effect was mainly related to the dose of RE metals and the duration of the incubation period in vitro.

## 7 PROSPECTS OF RE METALS IN BONE TISSUE ENGINEERING AND IMPLANTOLOGY

Bone regeneration is a complex process involving numerous factors, including the recruitment of progenitor cells, inflammation, early angiogenesis, and osteogenesis. RE nanomaterials have autophagy augmenting potential,^305^ whereas RE materials can induce autophagy, but there is no adequate evidence to prove this phenomenon. In order to intersect the role of RE materials induced autophagy would open up the new vistas in bone tissue engineering that can be applied to induce bone regenerative potential. RE nanomaterials contribute to sustaining mild ROS levels, which could modulate the redox state via autophagy in the MSCs to regulate the osteogenic processes effectively and eventually heal the bone defect.

Effective bone regeneration requires a continuous blood supply. Coordination between osteogenesis and angiogenesis is crucial for proper bone regeneration.^306^, ^307^, ^308^ Osteogenesis, angiogenesis, and osseointegration are essential for the successful restoration of bone mass. Tuning of such factors by designing with RE nano biomaterials is critical for bone tissue engineering.^46^, ^309^ In recent years, great attention has been drawn to coupling angiogenesis and osteogenesis to promote type H vessel formation. Type H vessels are a subtype of the capillary with high expression of CD31 and endomucin and promote osteogenesis. Type H vessels can actively direct bone formation by producing factors that stimulate the proliferation and differentiation of osteoprogenitors in the bone marrow.^81^, ^310^, ^311^, ^312^ Type H vessel‐inducing potential of rare‐earth‐based nanomaterials is not adequately studied. Understanding the role of RE‐based materials on type H vessel formation may open up new vistas in the bone tissue engineering field.

Osteocytes play a vital role in bone modeling, remodeling, and homeostasis. The primary function of osteocytes is to convert mechanical stimuli to biological signalings that regulate the functions of osteoblasts, osteoclasts, and immune cells. The effect of RE nanomaterials on osteocytes function has not been reported yet. Future research should focus on designing RE smart nano‐biomaterials that can modulate osteocyte function and promote bone regeneration. Similarly, immunomodulation regulation‐based bone tissue engineering is currently a hot research topic. Investigating the use of RE nanomaterials on spatio temporal control of macrophage polarization and infiltration of various immune cells, including T cells, B cells, and neutrophils, would lead to the applicability of nano immunoenginneering approaches in bone tissue.

The majority of cancer easily metastasized in the bone. The cancer metastasized to the bone is very difficult to treat and causes excessive osteolysis. Scientists are desperately trying to develop therapeutic approaches to treat cancer metastasized in bone and simultaneously rescue bone loss. RE nano‐biomaterials have bone regenerative and anti‐cancer properties. RE nano‐biomaterials has the potential to be used for in vivo imaging of cancer during diagnosis and treatment. Similarly, RE nano‐biomaterials have shown the potential for imaging the newly formed and osteoporotic bone. Therefore, the prospect should be focused on designing RE innovative nano‐biomaterials‐based targeted therapy that can treat cancer metastasized in bone, rescue metastasis‐induced bone loss, and simultaneously visualize the remaining cancer mass and newly formed bone.

In‐depth analysis of local and systemic adverse effects of RE‐nanobiomaterials in large animal models close to humans is another prospect that streamlines the clinical application of RE nano‐bio materials. The clinical complication can be minimized by using rare‐earth nanomaterials as a co dopant in new scaffold‐based mechanics like 3D printing or electrospinning.^313^, ^314^, ^315^ Electrospinning is the most practical and widely explored technique for synthetic membranous grafts. Biopolymers like collagen, silk, and synthetic polymers like polyethylene glycol (PEG) and poly(lactic acid) (PLLA) have been designed for tissue regeneration purposes.^316^, ^317^ Using the RE‐based nanomaterials with these techniques may yield a remarkable outcome in accelerating bone defect healing with structural and mechanical stability. RE materials doped electrospun or 3D‐printed scaffolds may aid to warrant the sustained release and site‐specific delivery of RE elements based on their physicochemical properties.

## 8 CONCLUSIONS

In summary, this review portrayed the technological innovations of RE‐based materials in bone tissue engineering. The intriguing features of RE materials such as biocompatibility, narrow band upconversion fluorescence property for deep tissue penetration, and excellent biological properties imply the promising potential of RE materials in biomedical applications. RE materials' antioxidant, immunomodulatory, angiogenic, and osteogenic properties could be utilized to fabricate cost‐effective bone grafts and implants. The mechanism of RE‐material‐based recruitment of progenitor cells, induction of early angiogenesis, and osteogenesis should be studied thoroughly. The role of RE materials on immunomodulation, autophagy machinery in osteoblasts and MSCs, type H vessel formation, osteocytes function, and endothelial regulations need to be thoroughly investigated. Nevertheless, dose optimization, mode of delivery, and local/systemic adverse effects should be thoroughly investigated in large animal models to guarantee the bench‐to‐bed translation. Overall, RE smart nano‐bio materials hold promising potential to substantiate the global demand for cost‐effective biomaterials for bone tissue engineering and implantology in the future.

## CONFLICT OF INTERESTS

All authors have no conflicts of interest.

## AUTHOR CONTRIBUTIONS

**Duraipandy Natarajan:** Conceptualization (lead); formal analysis (lead); investigation (lead); methodology (lead); writing – original draft (lead); writing – review and editing (lead). **Zhitong Ye:** Software (equal); visualization (equal). **Liping Wang:** Funding acquisition (equal); project administration (equal); resources (equal); supervision (equal). **Linhu Ge:** Funding acquisition (equal); project administration (equal); resources (equal); supervision (equal). **Janak Lal Pathak:** Conceptualization (lead); funding acquisition (lead); methodology (lead); project administration (equal); resources (equal); supervision (lead); validation (lead); writing – review and editing (lead).

### PEER REVIEW

The peer review history for this article is available at https://publons.com/publon/10.1002/btm2.10262.

## Data Availability

Data sharing not applicable to this article as no datasets were generated or analyzed during the current study
